# Y chromosome–linked UTY modulates sex differences in valvular fibroblast methylation in response to nanoscale extracellular matrix cues

**DOI:** 10.1126/sciadv.ads5717

**Published:** 2025-03-12

**Authors:** Rayyan M. Gorashi, Talia Baddour, Sarah J. Chittle, Nicole E. Félix Vélez, Michaela A. Wenning, Kristi S. Anseth, Luisa Mestroni, Brisa Peña, Peng Guo, Brian A. Aguado

**Affiliations:** ^1^ Shu Chien-Gene Lay Department of Bioengineering, University of California San Diego, La Jolla, CA 92093, USA.; ^2^ Sanford Consortium for Regenerative Medicine, La Jolla, CA 92037, USA.; ^3^ Department of Chemical and Biological Engineering, University of Colorado Boulder, Boulder, CO 80303, USA.; ^4^ BioFrontiers Institute, University of Colorado Boulder, Boulder, CO 80309, USA.; ^5^ Division of Cardiology, School of Medicine, Cardiovascular Institute, University of Colorado Denver Anschutz Medical Campus, 12700 E.19th Avenue, Bldg. P15, Aurora, CO 80045, USA.; ^6^ Division of Cardiology, Department of Medicine, University of Colorado Anschutz Medical Campus, 12700 E.19th Avenue, Bldg. P15, Aurora, CO 80045, USA.; ^7^ Department of Bioengineering, University of Colorado Denver Anschutz Medical Campus, Bioscience 2 1270 E. Montview Avenue, Suite 100, Aurora, CO 80045, USA.; ^8^ Nikon Imaging Center, Department of Cellular and Molecular Medicine, University of California San Diego, La Jolla, CA 92093, USA.

## Abstract

Aortic valve stenosis (AVS) is a progressive disease, wherein males more often develop valve calcification relative to females that develop valve fibrosis. Valvular interstitial cells (VICs) aberrantly activate to myofibroblasts during AVS, driving the fibrotic valve phenotype in females. Myofibroblasts further differentiate into osteoblast-like cells and produce calcium nanoparticles, driving valve calcification in males. We hypothesized that the lysine demethylase UTY (ubiquitously transcribed tetratricopeptide repeat containing Y-linked) decreases methylation uniquely in male VICs responding to nanoscale extracellular matrix cues to promote an osteoblast-like cell phenotype. Here, we describe a hydrogel biomaterial cell culture platform to interrogate how nanoscale cues modulate sex-specific methylation states in VICs activating to myofibroblasts and osteoblast-like cells. We found that UTY modulates the osteoblast-like cell phenotype in response to nanoscale cues uniquely in male VICs. Overall, we reveal a previously unidentified role of UTY in the regulation of calcification processes in males during AVS progression.

## Teaser

The Y chromosome modulates sex differences in valve cells during aortic valve calcification.

## INTRODUCTION

Aortic valve stenosis (AVS) is sexually dimorphic disease, where aortic valve (AV) tissue progressively undergoes fibro-calcification (*1*). AVS affects ~13% of the population aged over 75, and gender-based disparities exist with clinical management of AVS, given retrospective studies, suggesting that women are under recommended for valve replacements (*2*). Given that valve replacement patients are also at risk for increased co-morbidities after the procedure, a substantial need exists to identify nonsurgical-based strategies to delay the onset of AVS (*3*). Clinical research further suggests that the degree of fibro-calcification in the valve depends on biological sex, where males exhibit increased calcification relative to females (*4*, *5*). Given the sex dimorphisms observed in valve tissue during AVS onset, biological sex must be considered when identifying paths toward nonsurgical pharmacologic treatments for AVS.

Our collective understanding of the sex-specific fibro-calcification processes in valve tissue during AVS remains limited. Valvular interstitial cells (VICs) are the resident fibroblast-like cells in valve tissue that are known to regulate valve fibro-calcification (*4*). In early stages of AVS, VICs chronically activate to myofibroblasts and express α smooth muscle actin (αSMA) stress fibers (*6*). In moderate to late stages of AVS, myofibroblasts further differentiate into osteoblast-like valve cells (*7*, *8*). Osteoblast-like valve cells are characterized by the nuclear localization of runt-related transcription factor 2 (RUNX2) and contribute to the accumulation of stiff, spherical calcium phosphate nanoparticles (NPs) in valve tissue (*9*, *10*). Over time, NPs increase in size and abundance and further exacerbate valve fibro-calcification (*4*). We posit that the initiation of sex-specific AVS progression partially depends on VIC interactions with the valve extracellular microenvironment.

Calcific lesions observed in healthy and late-stage AVS tissue contain dense, mineralized spherical NPs of varying size in the valve extracellular microenvironment (*11*). Prior studies have shown that osteoblast-like cells experience altered cytoskeletal dynamics after culture on substrates containing stiff NPs (*12*). In particular, the NPs affected actin organization, disrupted microtubule networks, and reduced overall cell spreading. VICs interact with their surrounding microenvironment via transmembrane integrin receptors (*13*, *14*). Integrins are directly linked to the actin cytoskeleton and mediate cell response to external stimuli through alterations to the nuclear chromatin structure (*15*–*17*). Prior studies have also identified differences in chromatin structure of VICs from both healthy and diseased patients (*18*, *19*). As such, chromatin rearrangement in male and female VICs in response to nanoscale cues in the extracellular matrix is hypothesized to modulate sex-specific cellular phenotypes during valve fibro-calcification.

Furthermore, cellular responses to the extracellular microenvironment also depend on intracellular sex chromosome effects (*20*–*23*). Sex differences in AVS progression have been attributed to both patient-specific sex hormone levels and sex chromosome activity (*24*–*26*). To detangle the effects of sex hormones from sex chromosome activity, approaches using hydrogel biomaterials to recapitulate the valve extracellular matrix have been used to show that female VICs have increased myofibroblast activation due to genes that escape X chromosome inactivation (*27*, *28*). In males, Y chromosome–linked genes are known to modulate cardiac and inflammatory disease phenotypes. For instance, the hematopoietic loss of the Y chromosome in bone marrow cells was shown to accelerate mortality, age-related cardiac fibrosis, and reduced cardiac function (*29*). Furthermore, the ubiquitously transcribed tetratricopeptide repeat containing Y-linked (*UTY*) gene protects against pro-inflammatory cytokine production during pulmonary hypertension (*30*). Previous work has also shown hydrogels to be critical tools to understand sex-specific mechanotransduction in endothelial cells cultured in vitro (*31*, *32*). Our understanding of how VICs respond to nanoscale cues in the extracellular matrix will continue to harness in vitro culture tools to probe sex-specific cell-nanomaterial interactions and subsequent sex-specific effects on cell phenotypes.

Here, we interrogated how nanoscale matrix cues modulate the VIC-to-myofibroblast-to-osteoblast transition in males and females. We first performed single-cell sequencing on human valve tissue samples from patients with AVS, revealing up-regulation of pro-osteogenic genes and decreased expression of the Y chromosome–linked histone demethylase, known as *UTY* in male VICs. Next, we engineered a bioinspired hydrogel cell culture platform to probe how nanoscale matrix cues modulate VIC phenotypes via UTY. We have previously used hydrogel biomaterials to culture primary VICs isolated from AV leaflets and determined that sex chromosome–linked genes modulate sex differences in VIC phenotypes (*27*, *28*). In our current approach, we incorporated stiff, polystyrene NPs (PS-NPs) into a water-swollen cross-linked poly(ethylene glycol) (PEG)–norbornene (Nb) hydrogel to mimic nanoscale matrix cues in AV tissue. After culture on our PS-NP hydrogel, our results collectively show male-specific osteoblast-like responses to nanoscale stiffness cues. After *UTY* knockdown, we reveal up-regulation of genes involved in osteogenic differentiation, ultimately promoting calcification processes in VICs. We further validated our bioinspired hydrogel platform by comparing the in vitro male VIC transcriptome on PS-NP hydrogels with our single-cell transcriptomic dataset from human tissues. Overall, we have potentiated a role of the Y-linked UTY lysine demethylase in causing sex-specific effects that guide male VICs toward calcification in AVS.

## RESULTS

### Single-cell sequencing reveals distinct VIC populations in diseased human AVs

**Fig. 1.**
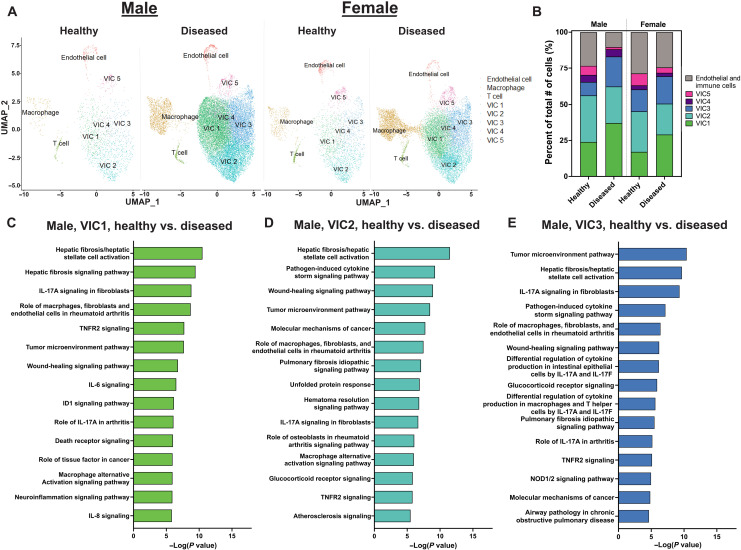
Single-cell sequencing and sex-separated analysis of human patients with AVS reveal clustering of VIC populations. (**A**) Uniform manifold approximation and projection (UMAP) clustering and cell population distribution in healthy and diseased patient samples for males and females. (**B**) Distribution of VIC and non-VIC population percentages in healthy and diseased patient samples for males and females. (**C** to **E**) Ingenuity pathway analysis for male (C) VIC1, (D) VIC2, and (E) VIC3. IL-17A, interleukin-17A; TNFR2, tumor necrosis factor receptor 2; IL-6, interleukin-6; ID1, inhibitor of differentiation 1; IL-8, interleukin-8; IL-17F, interleukin-17F; NOD1/2, nucleotide-binding oligomerization domain 1/2.

We first characterized sex-specific VIC populations in diseased AVs relative to healthy valves. We conducted our sex-separated analysis by combining publicly available data from patients with AVS and our own patient samples (table S1). After clustering, our uniform manifold approximation and projection (UMAP) plots show endothelial cells, macrophages, T cells, and five distinct VIC populations, in both male and female patients (Fig. 1A and fig. S1A). Cell populations were determined using conserved biomarkers and further identified by distinct biomarkers (figs. S1, B and C, S2, and S3). In both male and female patients, VIC population percentages significantly increase with disease and comprises most of detected cells in the AV tissue (Fig. 1B and fig. S4, A and B).

Ingenuity pathway analysis revealed significant associations with pathways related to pro-inflammatory and pro-fibrotic pathways, senescence, and interleukin-17 signaling for the male VIC1 population (Fig. 1C). The male VIC2 and VIC3 populations showed significant associations to pathways such as interleukin-17 signaling, tumor necrosis factor receptor–2 signaling, chondrocytes in inflammatory diseases, and Toll-like receptor signaling (Fig. 1, D and E). Male VIC4 and VIC5 showed significant associations to pathways involving senescence, death receptor signaling, and apoptosis signaling (fig. S5).

### Hydrogels containing PS-NPs mimic the valve microenvironment

**Fig. 2.**
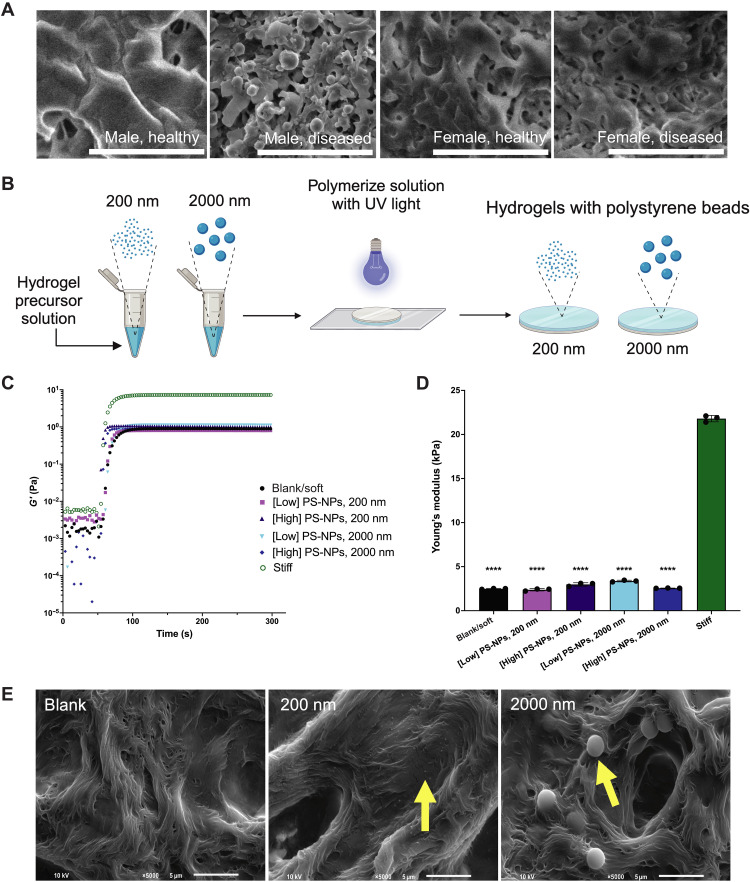
PS-NP hydrogels recapitulate nanoscale features in AV tissue ECM. (**A**) Scanning electron microscopy (SEM) images of male healthy and diseased and of female healthy and diseased human AV leaflets. Scale bars, 5 μm. (**B**) Schematic of process to incorporate PS-NPs into PEG-Nb hydrogel precursor solutions to create PS-NP hydrogels adhered to glass coverslips via ultraviolet (UV) polymerization. Figure was drawn with BioRender. (**C**) Rheological characterization of UV-mediated polymerization of blank/soft, stiff, and PS-NP hydrogels made with 200- and 2000-nm NPs (*n* = 3 representative measurements) at low and high concentrations (0.02 and 0.7 mg/ml, respectively). (**D**) Young’s modulus values for blank/soft, stiff, and PS-NP hydrogels (*n* = 3 gels). Significance determined via one-way analysis of variance (ANOVA; ****P < 0.0001 denoting statistical significance between hydrogel formulations). Means ± SD are shown. (**E**) SEM of blank and PS-NP hydrogels (200 nm and 2000 nm). Yellow arrows denote PS-NPs. Scale bars, 5 μm.

We next sought to engineer a hydrogel cell culture platform that recapitulates the valve matrix that contains nanoscale calcium phosphate particles. To determine the appropriate particle size scale, we characterized the nanoscale matrix features from age-matched male and female AV tissue from two healthy patients and two patients diagnosed with AVS. Using scanning electron microscopy (SEM), we visualized the accumulation of nanoscale calcium phosphate particles in AV tissue from male and female patients with AVS relative to healthy patients (Fig. 2A). After quantifying particle diameter, we observe that calcium phosphate particles in male diseased patients have the highest frequency distribution between 100 and 2000 nm. The largest particles from our severely stenotic male AVS patient sample measured 10,000 nm. In female diseased patients, we observe the highest frequency distribution between 50 and 1000 nm. In our severely stenotic female AVS patient sample, the largest particles measured 21,000 nm (fig. S6, A to C).

We next engineered PEG hydrogels containing 200- or 2000-nm-diameter PS-NPs as a strategy to mimic particle sizes within the particle size range observed in AV tissue from patients with early-stage AVS (Fig. 2B). We quantified hydrogel storage and elastic modulus using shear rheology (Fig. 2, C and D) and used atomic force microscopy to validate elastic modulus measurements (fig. S7A) and reveal the nanoscale surface topography of PS-NP gels (fig. S7, B and C). We determined that the elastic modulus (**E**) of our soft, blank hydrogel matrix formulation (**E** = 2.48 ± 0.05 kPa) does not significantly change with the incorporation of 200-nm PS-NPs (**E**_200-nm PS-NPs, low_ = 2.37 ± 0.12 kPa and **E**_200-nm PS-NPs, high_ = 2.98 ± 0.19 kPa) and 2000-nm PS-NPs (**E**_2000-nm PS-NPs, low_ = 3.36 ± 0.09 kPa and **E**_2000-nm PS-NPs, high_ = 2.55 ± 0.02 kPa), relative to our stiff hydrogel formulation (**E** = 21.80 ± 0.36 kPa). We also used SEM to confirm PS-NP presence on the surface of the hydrogel matrix (Fig. 2E) and confocal imaging to visualize the distribution of the PS-NPs within the top ~90 μm of the hydrogel matrix (fig. S8).

### Sex-specific myofibroblast activation and osteoblast-like differentiation on NP hydrogels

**Fig. 3.**
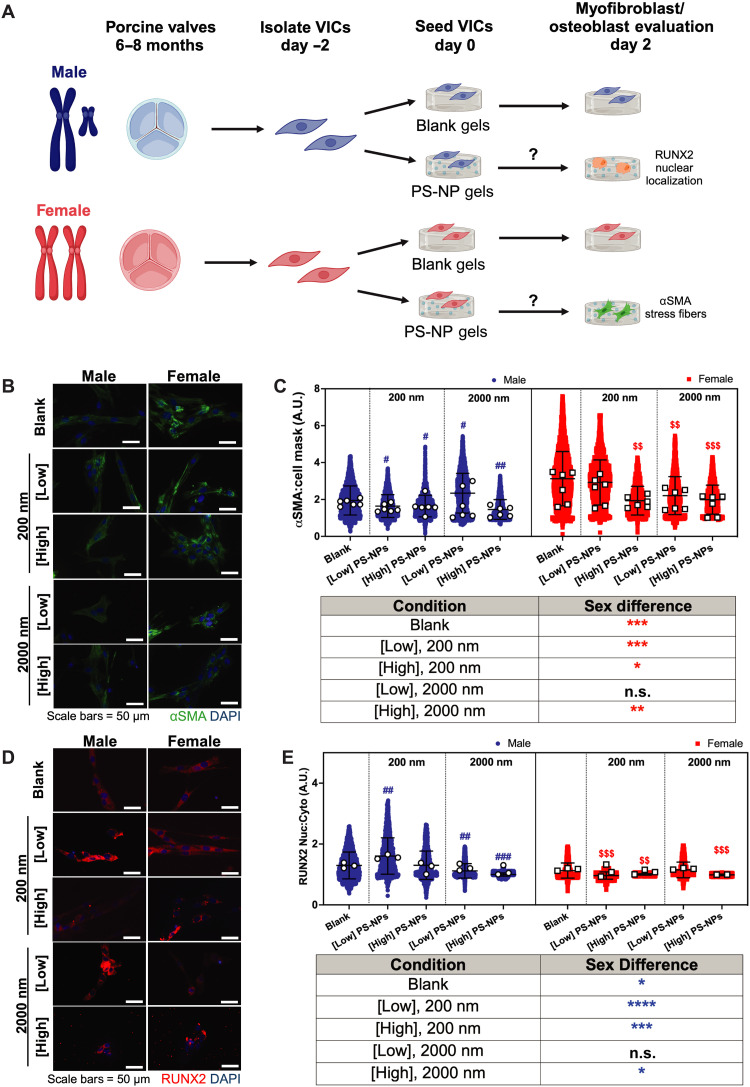
Male and female VICs exhibit sex-specific myofibroblast and osteoblast-like phenotypes. (**A**) Schematic of experiments assessing myofibroblast activation with αSMA and osteoblast-like differentiation with runt related (RUNX2). Figure was drawn with BioRender. (**B**) Representative confocal microscopy images of αSMA stress fibers (green) and nuclei (blue). Scale bars, 50 μm. (**C**) Normalized αSMA expression for male and female VICs cultured on blank hydrogels with no PS-NPs, 200-nm PS-NPs, or 2000-nm PS-NPs at low and high concentrations (0.02 and 0.7 mg/ml, respectively). (**D**) Representative confocal microscopy images of RUNX2 expression (red) and nuclei (blue). Scale bars, 50 μm. (**E**) RUNX2 nuclear to cytoplasmic intensity ratios of male and female VICs cultured on blank hydrogels with no PS-NPs, 200-nm PS-NPs, or 2000-nm PS-NPs at low and high concentrations (0.02 and 0.7 mg/ml, respectively). Significance for [(C) and (E)] was determined via Cohen’s *d* test (*d < 0.2, **d < 0.5, ***d < 0.8, and ****d < 1.4 denoting significance between biological sex in the provided table; blue asterisk color indicates significant increase in male groups; #*d* < 0.2, ##*d* < 0.5, ###*d* < 0.8, and ####*d* < 1.4 denoting significance relative to male blank with no particles; $*d* < 0.2, $$*d* < 0.5, $$$*d* < 0.8, and $$$$*d* < 1.4 denoting significance relative to female blank with no PS-NPs). White circles and squares represent the mean for each hydrogel replicate for males and females, respectively. Blue circles and red squares represent individual cells as technical replicates for males and females, respectively. A.U., arbitrary units; n.s., not significant.

To test the effect of PS-NPs on sex-specific valve cell phenotypes, we isolated VICs from porcine AV tissue for culture on our PS-NP hydrogels (Fig. 3A). First, using αSMA stress fibers as a marker of myofibroblast activation, we observed female VICs having significantly higher levels of myofibroblast activation on both the blank hydrogel and PS-NP hydrogel conditions, compared to male VICs (Fig. 3, B and C). Second, we evaluated the nuclear localization of the transcription factor RUNX2 as a marker of osteoblast-like differentiation. Male VICs displayed significantly higher levels of RUNX2 nuclear localization on the blank hydrogel and most PS-NP hydrogel conditions, compared to female VICs (Fig. 3, D and E). Collectively, our results provide evidence that male and female VICs exhibit sex-specific differences in myofibroblast and osteoblast-like phenotypes in response to PS-NPs in culture. Although we detected these sex-specific phenotypes in response to PS-NP hydrogels, we did not observe consistent dose-dependent or PS-NP size-dependent trends.

### Sex-specific methylation and acetylation states on PS-NP hydrogels

**Fig. 4.**
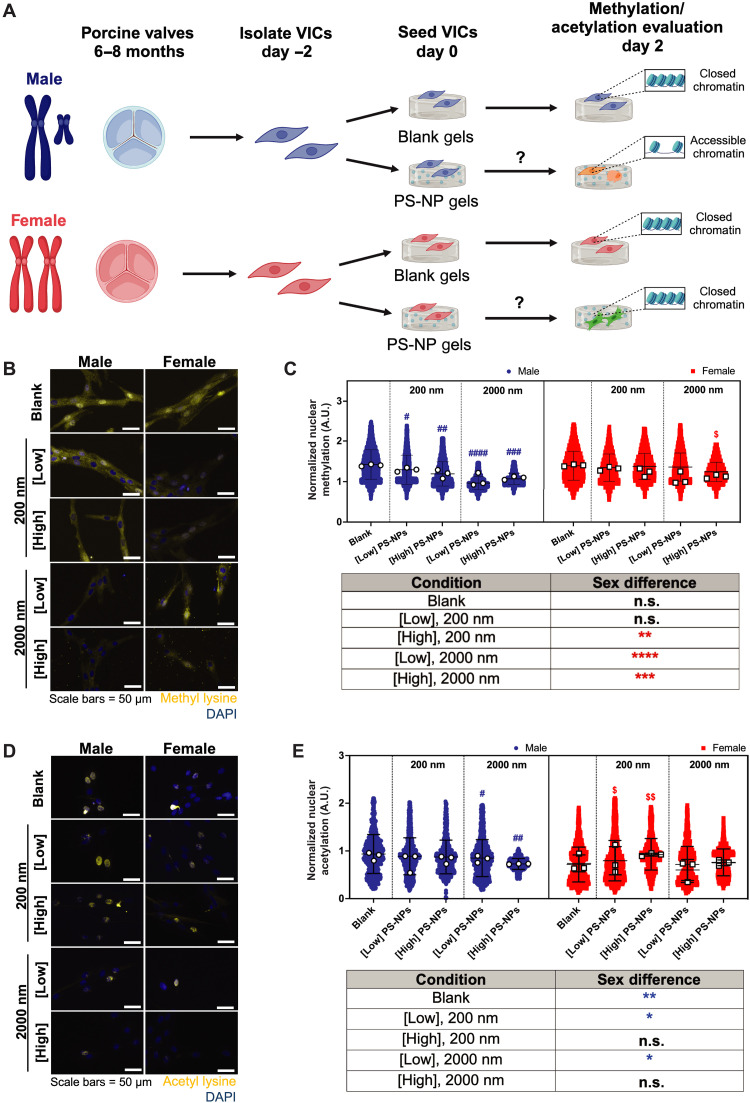
Male and female VICs exhibit sex-specific changes in global methylation and acetylation states. (**A**) Schematic describing experimental workflow for assessing sex-specific methylation and acetylation states in VICs cultured on PS-NP gels. Figure was drawn with BioRender. (**B**) Representative confocal microscopy images of methylated lysine (yellow) and nuclei (blue). Scale bars, 50 μm. (**C**) Normalized methylation in male and female VICs cultured on blank hydrogels with no PS-NPs, 200-nm PS-NPs, or 2000-nm PS-NPs at low and high concentrations (0.02 and 0.7 mg/ml, respectively). (**D**) Representative confocal microscopy images of acetylated lysine (yellow) and nuclei (blue). Scale bars, 50 μm. (**E**) Normalized acetylation in male and female VICs cultured on control hydrogels with no PS-NPs, 200-nm PS-NPs, or 2000-nm PS-NPs at low and high concentrations (0.02 and 0.7 mg/ml, respectively). Significance for [(C) and (E)] was determined via Cohen’s *d* test (*d < 0.2, **d < 0.5, ***d < 0.8, and ****d < 1.4 denoting significance between biological sex in the provided table; blue asterisk color indicates significant increase in male groups; #*d* < 0.2, ##*d* < 0.5, ###*d* < 0.8, and ####*d* < 1.4 denoting significance relative to male blank with no particles; $*d* < 0.2, $$*d* < 0.5, $$$*d* < 0.8, and $$$$*d* < 1.4 denoting significance relative to female blank with no PS-NPs). White circles and squares represent the mean for each hydrogel replicate for males and females, respectively. Blue circles and red squares represent individual cells as technical replicates for males and females, respectively.

Recognizing that epigenetic modifications drive cellular phenotype plasticity via alterations to methylation and acetylation states in the nucleus (*33*), we sought to characterize sex-specific epigenetic states in VICs in response to nanoscale stiffness cues. To understand cell-matrix interactions that modulate transcriptional activity, we sought to investigate methylation states in VICs cultured on PS-NP hydrogels (Fig. 4A). Male VICs displayed reduced tri-methylation across most hydrogel conditions relative to female VICs (Fig. 4, B and C). We observed dose-dependent and PS-NP size-dependent changes in male VIC methylation after culture on PS-NP hydrogels. Next, we determined acetylation states in VICs cultured on PS-NP gels, hypothesizing that male VICs would have increased acetylation to drive expression of osteoblast-associated genes. We observed a unique trend in the males, where only larger particles affected the acetylation state (Fig. 4, D and E). Male VICs also displayed significantly higher acetylation states (Fig. 4D) in most of our blank and PS-NP hydrogel conditions, when compared to female VICs (Fig. 4E). Our evidence suggests that male-specific epigenetic modifications may regulate male-specific osteoblast-like differentiation on PS-NP hydrogels.

### UTY modulates male-specific VIC phenotypes in vivo and in vitro

We next hypothesized that epigenetic modifiers coded on the Y chromosome may contribute to male-specific VIC phenotypes in vivo and in vitro. Specifically, we posit that a Y-linked demethylase may play a key role in regulating VIC response to nanoscale cues in the extracellular matrix. We interrogated the role of the Y-linked global demethylase UTY in regulating VIC phenotype in vivo and variable methylation in cells cultured on PS-NP hydrogels.

**Fig. 5.**
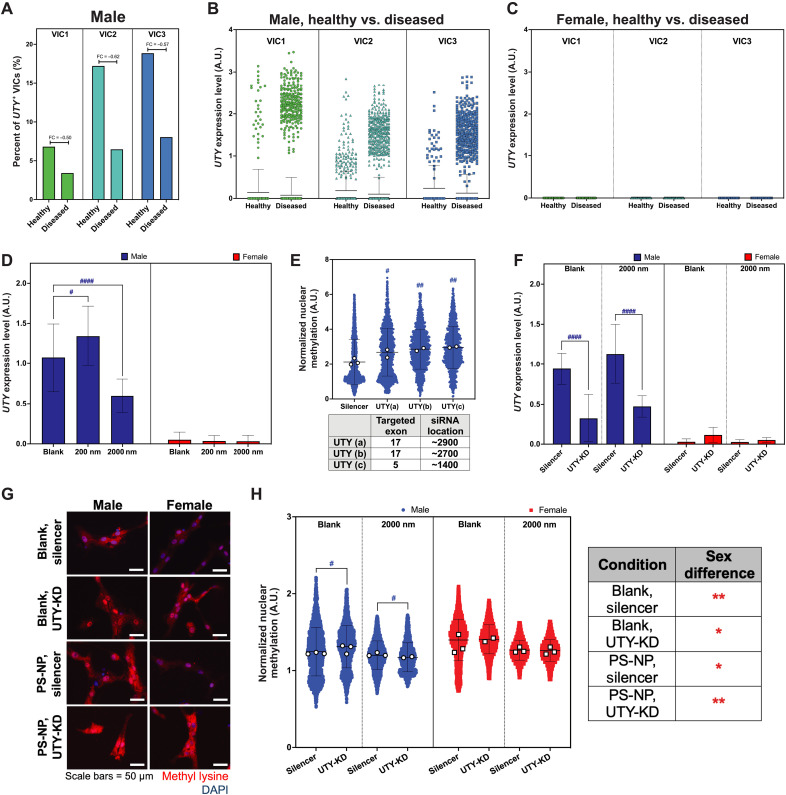
Y-linked *UTY* modulates male-specific methylation states in VICs in vivo and in vitro. (**A**) Fold change (FC) of *UTY*^+^ VICs in diseased human male VIC populations VIC1-3 relative to healthy male VICs. Each percentage is shown relative to the total number of VICs in each specified subpopulation. (**B** and **C**) *UTY* relative mRNA expression in VIC populations 1 to 3 in (B) males and (C) females. (**D**) Real-time quantitative polymerase chain reaction (RT-qPCR) for relative expression of *UTY* in male and female VICs cultured on blank, 200-nm, and 2000-nm PS-NP hydrogels. (**E**) Normalized methylation for male VICs with three *UTY*-targeting siRNAs for exon regions 17 and 5. (**F**) RT-qPCR to validate partial silencing of *UTY* in male VICs after introduction of siRNA vector. (**G**) Representative confocal microscopy images of methylated lysine (red) and nuclei (blue). Scale bars, 50 μm. (**H**) Normalized methylation in male and female VICs cultured on blank hydrogels with no PS-NPs and 2000-nm PS-NPs. Significance determined via Cohen’s *d* test (*d < 0.2 and **d < 0.5 denoting significance between biological sex in the provided table; blue asterisk color indicates significant increase in male groups; #*d* < 0.2, ##*d* < 0.5, ###*d* < 0.8, and ####*d* < 1.4 denoting significance relative to male silencer; $*d* < 0.2, $$*d* < 0.5, $$$*d* < 0.8, and $$$$*d* < 1.4 denoting significance relative to female silencer). White circles and squares represent the mean for each hydrogel replicate for males and females, respectively. Blue circles and red squares represent individual cells as technical replicates for males and females, respectively.

We first evaluated *UTY* expression in diseased AVS valve tissue using our single-cell sequencing human AV datasets. In males, VIC1, VIC2, and VIC3 displayed a higher count of *UTY^+^* cells compared to VIC4 and VIC5 (fig. S9A). The overall percentage of VICs expressing *UTY* decreases with disease (Fig. 5A), and subsets of VICs within each population have increased *UTY* expression levels in disease, indicating heterogeneity in *UTY* expression (Fig. 5B). Female VICs did not display detectable *UTY* expression in both healthy and diseased samples, as expected (Fig. 5C and fig. S8B). We also characterized expression of the ubiquitously transcribed tetratricopeptide repeat, X chromosome (*UTX*) gene, revealing heterogeneous expression in both male and female VICs (fig. S9, C and D).

To interrogate the role of *UTY* in regulating response to nanoscale stiffness cues in vitro, we used a small interfering RNA (siRNA) targeting the *UTY* gene to transcriptionally silence *UTY* while VICs are cultured on PS-NP hydrogels. First, we validated that *UTY* expression is male-specific on blank and PS-NP hydrogels using real-time quantitative polymerase chain reaction (RT-qPCR) (Fig. 5D). Next, we optimized our *UTY*-targeting siRNA location and targeted exon. We found that *UTY* siRNA that targets exon 5, or *UTY* (c), resulted in the largest increase in global methylation after siRNA treatment relative to the silencer negative control on a glass substrate (Fig. 5E). For subsequent experimentation on blank and PS-NP hydrogels, we used *UTY* (c).

After optimizing our *UTY* siRNA–knockdown (*UTY*-KD) procedure, we next used RT-qPCR to validate that we significantly reduced relative *UTY* gene expression uniquely in male VICs cultured on PS-NP hydrogels (Fig. 5F). Female VICs did not show significant *UTY* expression in all our conditions, further validating a male-specific reduction in *UTY* expression. As another means of validation, we used immunofluorescence to detect global methylation in male and female VIC nuclei (Fig. 5G). As hypothesized, *UTY*-KD in male VICs led to a significant increase in methylation on blank hydrogels (Fig. 5H). Female VICs did not increase methylation after *UTY*-KD on blank gels. On PS-NP gels, *UTY*-KD did not affect methylation in female VICs. Across each condition, female VICs also maintained higher levels of methylation. Together, we suggest that *UTY* modulates male-specific methylation states in VICs cultured on our engineered hydrogels.

### Transcriptomic analyses unveil up-regulation of pro-osteoblast pathways after *UTY* knockdown in porcine VICs on PS-NP hydrogels

**Fig. 6.**
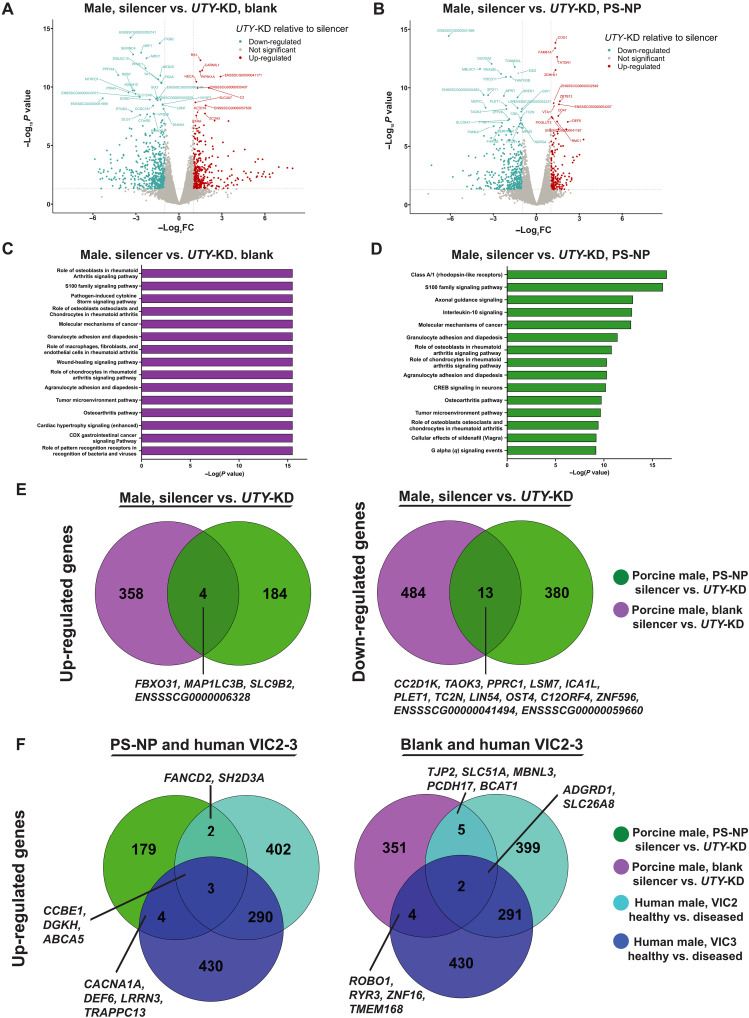
Transcriptomics analyses in porcine male VICs after *UTY*-KD unveils osteoblast-associated pathways. (**A** and **B**) Bulk mRNA sequencing shows differentially expressed genes (DEGs) in male porcine VICs (blue, down-regulated; red, up-regulated; gray, not significant) in (A) blank and (B) PS-NP hydrogels using DESeq2. (**C** and **D**) Ingenuity pathway analysis of porcine VICs after *UTY*-KD on (C) blank and (D) PS-NP hydrogels [log_2_ fold change (log_2_FC) > 1, log_2_FC < −1, and *P* value < 0.05]. (**E**) Venn diagrams of up-regulated and down-regulated DEGs in porcine VICs cultured on blank and PS-NP hydrogels after *UTY*-KD. (**F**) Venn diagrams of up-regulated genes in common between porcine and human transcriptomics datasets.

Next, we hypothesized that *UTY*-KD would up-regulate signaling networks associated with osteoblasts uniquely in male VICs. We performed bulk mRNA sequencing to understand the sex-specific transcriptomic responses to *UTY*-KD, given our prior observations revealing sex-specific differences in myofibroblast and osteoblast-like phenotypes, in addition to sex-specific methylation and acetylation states. In both blank and PS-NP gels, male VICs displayed a larger number of differentially expressed genes (DEGs) compared to female VICs (Fig. 6, A and B, and fig. S10, A and B). On blank hydrogels, male and female VICs had 861 and 221 genes differentially expressed, respectively. On PS-NP hydrogels, male and female VICs had 583 and 336 genes differentially expressed, respectively.

Ingenuity pathway analysis of male VICs with *UTY*-KD on blank (Fig. 6C) and PS-NP gels (Fig. 6D) showed strong associations to pathways related to osteoblasts and chondrocytes in inflammatory diseases, osteoarthritis signaling, and interleukin-4, interleukin-13, and interleukin-17 signaling, among others. Female VICs did not show any associations to pathways directly related to osteoblasts (fig. S10, C and D). Next, we compared the significant up-regulated and down-regulated DEGs in male VICs on both hydrogel substrates after *UTY*-KD (Fig. 6E). We observed 13 down-regulated genes and four up-regulated genes in common between blank and PS-NP gels in male VICs. These data suggest that *UTY* knockdown modulates genes relevant to cellular responses to microenvironmental cues.

Next, we compared our in vitro porcine transcriptomic dataset with our human AV single-cell sequencing dataset to assess how our PS-NP hydrogel system recapitulates VIC differentiation processes in vivo. We compared genes up-regulated after *UTY* knockdown in male porcine VICs to genes up-regulated in diseased human male VICs with at least twofold reduction in *UTY* expression (VIC2 and VIC3). VIC1 was not included in this comparison because of its strong associations with inflammatory signaling processes, relative to VIC2 and VIC3. We observed a subset of common genes between our human VICs and porcine VICs on both blank and PS-NP hydrogels (Fig. 6F and figs. S11 and S12). Genes associated with pro-osteogenic differentiation and calcium homeostasis, such as *DGKH* (*34*, *35*), *DEF6* (*36*, *37*), and *LRRN3* (*38*), were commonly identified between the porcine VICs with *UTY* knockdown and human data. We also compared up-regulated genes in porcine VICs to known epigenetic modifiers (*39*) and identified candidate osteogenic-associated transcriptional regulators. We observed *SUPT3H*, which has been linked to RUNX2-induced osteogenic differentiation (*40*–*42*), in the human male VIC2-3 populations and the porcine male PS-NP condition, but not the blank hydrogel condition (table S7). Together, the human VIC analysis supports our PS-NP hydrogel system in the recapitulation of key aspects of stiffness-induced osteogenic differentiation via modulating *UTY* expression levels.

## DISCUSSION

**Fig. 7.**
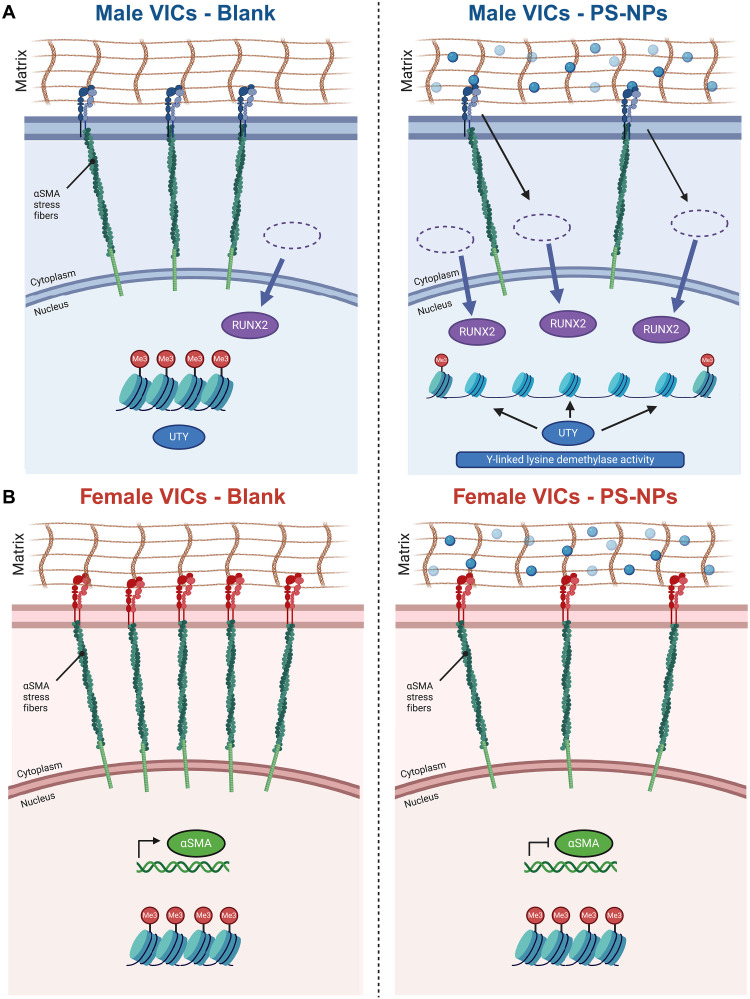
Proposed mechanism for sex-specific transcriptomic regulation in the presence and absence of nanoscale stiffness cues. At baseline conditions, male VICs maintain higher levels of RUNX2 nuclear localization, while females maintain higher levels of αSMA stress fibers. (**A**) In the presence of PS-NPs, we observed increased demethylation in male porcine VICs, whereas (**B**) we only observed decreases in αSMA stress fiber presence in female porcine VICs.

Here, we highlight that the Y chromosome modulates male-specific VIC phenotypes to nanoscale cues in the extracellular matrix relative to female VICs (Fig. 7). Using PEG hydrogels with PS-NPs as a platform for cell culture, we show that the protein-coding gene *UTY* may modulate male-specific demethylation in VICs during calcification in AVS. Recent studies have described the significant role of the mosaic loss of the Y chromosome (mLOY) in male-specific cardiovascular disease progression (*29*, *43*, *44*). mLOY occurs as an aging-related mutation in males, although the heterogeneity in mLOY in varying cell types and relation disease progression is not well understood. In AVS, male patients who experience mLOY have a pro-fibrotic gene expression signature in monocytes, leading to increased patient mortality after transcatheter AV replacement (*43*). Furthermore, UTY is also known to protect against pro-inflammatory phenotypes in lung tissue during pulmonary hypertension (*30*). Our results provide additional evidence supporting a protective role of the Y chromosome in disease progression, specifically valve calcification. Our results also further corroborate prior efforts implicating Y chromosome–linked genes, including *UTY*, as key modulators of male-specific cell phenotypes (*29*, *30*, *43*). Together, our work here provides a hydrogel cell culture platform that enables the interrogation of Y chromosome–linked genes and their impact on male-specific cellular phenotypes.

To the best of our knowledge, our study is the first to directly show that *UTY* influences male-specific VIC myofibroblast activation and osteoblast-like differentiation in response to extracellular matrix cues. Primary VICs from porcine sources have long been used to evaluate myofibroblast activation and osteogenic differentiation processes in AVS in response to different bulk substrate stiffnesses (*31*, *45*–*51*). For example, in response to tissue culture plastic (elastic modulus, *E* ~ 1 GPa), VICs automatically activate to myofibroblasts through the expression of αSMA stress fibers (*27*). As an alternative approach, PEG hydrogel platforms have been used to study sex-specific modulation of myofibroblast activation and osteoblast differentiation states. For example, previous work has shown that VICs cultured in two-dimensional (2D) or 3D PEG-Nb hydrogels in the ~1- to ~40-kPa stiffness range recapitulates sex-specific myofibroblast phenotypes observed in valve tissue (*27*, *28*). Using a similar hydrogel system, we incorporated NPs into the hydrogel matrix to mimic nanoscale features in valve tissue, as observed in our SEM images and previous work (*27*, *28*, *51*). Our work suggests that RUNX2 nuclear localization in VICs responding to nanoscale features in tissue may modulate a male-specific bias toward the initiation of calcification processes.

Our study also showed that *UTY* alters the VIC transcriptome on hydrogels via modulations in tri-methylation. Epigenetic modifications can occur in response to an external mechanical stimulus (*18*, *19*), which alters the conformation of cell surface receptors and cytoskeletal network tied to the nucleus (*23*). In the case of valve myofibroblasts, VICs cultured on hydrogels with varied stiffness reveal distinct chromatin structures similar to healthy and diseased valve tissue (*18*, *19*). Building upon this work, we show that male specific methylation and acetylation states may be modulated via UTY demethylase activity. When evaluating methylation in males, we observed a consistent trend in decreased methylation correlating with particle size and density, indicating that male myofibroblasts interact differently with nanoscale features in an extracellular microenvironment relative to female myofibroblasts. Female VICs did not show a decrease in methylation when interacting with nanoscale cues but did reduce methylation with larger NPs, indicating that other demethylases may participate in female-specific cell-particle interactions. A previous study showed that female induced pluripotent stem cells cultured with NPs experienced compromised differentiation processes via disordered X chromosome inactivation, although female-specific effects of NPs on pluripotent versus somatic cell cultures have yet to be compared (*52*). Future efforts will need to assess the role of other demethylases in common between XY and XX cells or potential activity of X-linked demethylases (e.g., *UTX* and *KDM5C*) to supplement our observations in male VICs.

Our transcriptomics analyses revealed *UTY* as a modulator of osteogenic-associated genes relevant to calcification. We found that reduced *UTY* expression in vitro led to the up-regulation of numerous pathways broadly associated with osteoblast signaling, inflammation, and wound healing, which mirror up-regulated pathways in human male VIC2-3 populations with reduced *UTY* expression. Of note, we found that epigenetic modifiers *SUPT3H*, *DEF6*, and *LRRN3* were uniquely up-regulated in the porcine PS-NP *UTY*-KD condition along with human male VIC2-3. Previous work has shown that *SUPT3H* promotes H3 acetylation and subsequent Runx2 transcription (*40*–*42*), promoting osteoblast differentiation. Prior studies have also linked demethylation to accelerated osteogenic differentiation processes via *DEF6* (*36*, *37*) and *LRRN3* (*38*). Our analysis also identified up-regulation of matrix remodeling and calcification-associated genes in VICs with *UTY* knockdown, including *CCBE1* (*53*) and *DGKH* (*34*, *35*). We also identified several pro-fibrotic genes and genes involved in myofibroblast activation and age-related cardiovascular diseases, including *MBNL3* (*54*) and *ROBO1* (*55*). Collectively, we posit that *UTY* may promote male-specific calcification processes in the human AV via multiple gene signaling networks. Our work also provides a stepping stone to future validation studies to further understand how sex chromosomes and the extracellular microenvironment synergistically contribute to somatic cell phenotypes in health and disease.

There are several limitations of the PS-NP hydrogel platform described in our study. First, our study did not fully decouple the effects of sex hormone imprinting from sex chromosomes (*56*–*58*), as only our male pigs were gonadectomized at birth. We envision future work where VICs from gonadectomized males and female animal models can be used to further study the effects of the sex chromosome on VIC phenotype. Second, mechanical and biochemical cues synergistically modulate the VIC to myofibroblast to osteoblast-like cell transition during AVS. Future cell culture platforms can be adapted to include inflammatory factors secreted from macrophages to evaluate epigenetic modifications in response to inflammatory cytokines and nanoscale stiffness cues. Third, our study also evaluated cell phenotypes after 2 days in culture. Acknowledging that AVS is a time-dependent progressive disease, we suggest future studies where longer time points are used to evaluate sex-specific chromatin structures (e.g., after days to weeks in culture) as VICs acquire a more permanent myofibroblast phenotype reflective of diseased valve tissue. Fourth, future efforts to use spherical calcium phosphate particles instead of PS-NPs may be used to further enhance the physiological relevance of the in vitro culture platform and evaluate effects of *UTY* and other Y-linked genes on calcification (*59*).

Together, our study implicates the importance of sex chromosome-linked genes in the progression of disease-driving phenotypes in AVS. Our bioinspired hydrogel platform provides evidence that nanoscale cues in the extracellular matrix influence sex-specific phenotypes in VICs and broadly suggests that biomaterials are useful tools to answer research questions related to sex-specific biology (*60*, *61*). We posit that Y-linked epigenetic regulators, such as UTY, regulate male-specific osteoblast-like phenotypes, contributing to earlier onset of valvular calcification in males. Future studies will explore the interplay between mechanical stimuli and sex-specific epigenetic regulation in males and females.

## MATERIALS AND METHODS

### NP hydrogel fabrication

Eight-arm, 40-kDa PEG-Nb was synthesized as previously described (*62*). Hydrogel precursor solutions were made mixing together 4% (w/v) PEG-Nb, 5-kDa PEG-dithiol cross-linker (2.96 mM, JenKem), cysteine-arginine-glycine-aspartic acid-serine (CRGDS) peptide (2 mM, Bachem), phosphate-buffered saline (PBS; Gibco, catalog no. 14-190-250), and photoinitiator lithium phenyl-2,4,6-trimethyl-benzoylphosphinate (1.7 mM; Sigma-Aldrich, catalog no. 900889). To incorporate NPs into hydrogel precursor solutions, we used 1% w/v aqueous solutions of 200-nm (Nanocs, catalog no. PS01-200) or 2000-nm (Nanocs, catalog no. PS01-2u) PS-NPs instead of PBS in the hydrogel precursor formulation. PS-NP aqueous solution was vortexed for 1 min before incorporation to hydrogel precursor solution. All hydrogels were made using a 0.99:1 thiol-to-ene ratio. Vapor deposition was used to functionalize 12-mm glass coverslips (Mercedes Scientific, catalog no. MER0012) and 25-mm glass coverslips (Mercedes Scientific, catalog no. MER0025) with thiol groups to allow for hydrogel adhesion. Briefly, coverslips were placed in an autoclave jar along with 100 μl of mercaptopropyltrimethoxysilane (Sigma-Aldrich, catalog no. 175617) in a 60°C oven overnight for thiol functionalization. Glass slides were coated with Sigmacote (Sigma-Aldrich, catalog no. SL2) to create a hydrophobic glass surface during hydrogel fabrication. Either 11.3 or 65 μl of hydrogel precursor solution was pipetted onto the Sigmacote glass slide and covered with a 12- or 25-mm coverslip, respectively. The hydrogels were photopolymerized at 4 mW/cm^2^, removed with a razor blade, sterilized with 5% (v/v) isopropyl alcohol/PBS solution, and rinsed three times with PBS. After the final PBS rinse, cell culture medium containing Media 199 (Gibco, catalog no. 11043023) supplemented with 1% fetal bovine serum (Gibco, catalog no. 16000069) and 50 U of penicillin and streptomycin (0.05 mg/ml) (Sigma-Aldrich, catalog no. P4458) was added to the hydrogels for overnight swelling before cell culture. For PS-NP confocal microscopy validation experiments, PS-NP hydrogels were prepared as described above using fluorescein isothiocyanate (FITC)–labeled 2000-nm PS-NPs (Nanocs, catalog no. PS2u-FC-1) and a Cy5 PEG-Thiol (Nanocs, catalog no. PG2-S5TH-5 k) instead of CRGDS. FITC-labeled PS-NPs were batch sonicated for 10 min before hydrogel fabrication to prevent aggregation. The top ~90 μm of the blank/soft and PS-NP hydrogel surface was imaged using a *Z*-stack at 0.3 μm per slice (fig. S8).

### Rheology

Hydrogels containing NPs were prepared as described above for rheological measurements. Storage (**G′**) and loss (**G″**) moduli were measured using a DHR-3 rheometer (TA Instruments) with an 8-mm parallel plate geometry. Oscillatory shear rheology was used with an amplitude of 1% and frequency of 1 Hz (*27*). To obtain the elastic modulus (**E**), the following formula was used: **E** = 2 * **G′** (1 + **v**), where **v** is Poisson’s ratio. We used a value of **v** = 0.5, assuming **G′** >>> **G″** for viscoelastic hydrogels.

### Human aortic valve tissue digestion and tissue preservation

Human AV tissue samples were obtained from the National Disease Research Interchange with UC San Diego Institutional Review Board approval (UCSD IRB 804209). AVs were excised from human hearts (postmortem interval < 48 hours) and washed with Earle’s balanced salt solution (EBSS; Gibco, catalog no. E2888). Valve leaflets without evidence of calcification were categorized as “healthy.” We also categorized mid- and late-stage AVS patient samples as “diseased” to better represent the heterogeneity of disease presentation in patients (table S1). Briefly, valve leaflets were then digested with Liberase (Roche, catalog no. 05401119001) for 30 min before quenching with medium for centrifugation. The resulting cell pellet was treated with Red Blood Cell Lysis Buffer (Invitrogen, catalog no. 00433357) for 1 min before quenching with medium and centrifugation. Cells were counted manually with a hemocytometer before subsequent preparation for single-cell sequencing. Valve leaflets were also placed in 4% paraformaldehyde and stored at 4°C for paraffin embedding, sectioning, and subsequent SEM.

### SEM on hydrogels and human valve tissue and quantification of particle size distribution

For SEM of hydrogels, samples were prepared on coverslips, frozen in liquid nitrogen, and lyophilized. SEM was performed using a JSM-6010LA instrument (JEOL Ltd., CU Anschutz) using the manufacturer’s settings. For SEM on human valve tissue, samples were fixed, paraffin embedded, and sectioned onto glass slides. Samples were sputter coated with iridium for 30 s at 75% power and imaged using a FEI Quanta FEG 250 on the manufacturer’s settings. Average calcium particle size was determined from two biological replicates for each sex in the disease condition using ImageJ as shown in fig. S6A.

### Atomic force microscopy

For atomic force microscopy measurements in fig. S7A, hydrogels mounted on coverslips were immersed in PBS and analyzed using a JPK NanoWizard 4a instrument (JPK Instruments). Hydrogels were probed using CB3 cantilevers with triangular tips (Nanosensors, catalog no. qp-BioAC-CI) until an initial force of 1 nN was reached. Calibration of the cantilever was made, applying the thermal noise method. Force spectroscopy was performed at each node of a square grid distributed over a 20 μm–by–20 μm grid. Force-displacement (*F*-*d*) data were collected using a peak load of 5 nN at a displacement rate of 10 μm/s. Force-deformation (*F*-δ) data were calculated using *F*-*d* curves by subtracting the initial cantilever deflection. To determine Young’s modulus *E* at each node, the loading portion of each *F*-δ curve was fit to an analytical model for a rigid conical tip in contact with an elastic half-space, i.e., *F* = (2/π)(*E*/1 − ν^2^)(tanα)δ^2^, using angle α determined from SEM images, an assumed Poisson’s ratio for the hydrogel (ν = 0.5), and *E* as the sole fitting parameter. For atomic force microscopy measurements in fig. S7 (B and C), blank/soft and PS-NP hydrogels were analyzed using a Park NX20 instrument (Park Systems) and probed using cantilevers with rectangular tips (NanoAndMore, USA, catalog no. NSC36). Hydrogel samples were probed using PinPoint Nanomechanical mode over a 5 μm–by–5 μm grid at 64-bit resolution. Data were corrected using the Johnson-Kendall-Roberts (JKR) adjustment and visualized using Park Systems XEI software (v5.2.4).

### Porcine VIC isolation and culture

AVs were dissected from 5- to 8-month-old male or female pigs (Midwest Research Swine) to obtain VICs. For each isolation, four biological replicates per sex were pooled together from the porcine AV leaflets. The data in Figs. 3 to 6 are comprised from a total of four VIC isolations. For each experiment, at least two batches of VIC isolations were used. Two VIC isolations were used to generate the data in Figs. 3 and 6, a third isolation used for Fig. 4, and a fourth isolation used for Fig. 5. After dissection, AV leaflets were submerged and rinsed in EBSS containing 50 U of penicillin and streptomycin (0.05 mg/ml). Next, leaflets were digested in EBSS containing 250 U of collagenase type 2 (Worthington Biochemical, catalog no. LS004176) for 30 min at 37°C under constant agitation at 100 rpm. After, collagenase solution was aspirated from the AV leaflets to remove endothelial cells. A secondary digestion with fresh collagenase solution was performed for 60 min under agitation at 100 rpm. To collect VICs, the remaining tissue was scraped against a 100-μm cell strainer, washed with VIC medium, and then centrifuged at 400*g* for 10 min. The supernatant was aspirated and replaced with fresh VIC expansion medium, composed of Media 199 supplemented with 15% FBS and 50 U of penicillin and streptomycin ( 0.05 mg/m). Freshly isolated VICs were cultured in VIC expansion medium with medium changes every other day until confluency. VICs were frozen down at passage 1. All experiments used passage 2 VICs at 35,000 cells/cm^2^. To reduce proliferation during experimentation, passage 2 VICs were cultured in VIC medium containing 1% FBS.

### siRNA gene knockdown

Gene knockdowns in the porcine VICs were conducted using siRNAs. We used *UTY* siRNA (Ambion, catalog no. 4392421, ID s14740). On day 0, cells were seeded on coverslips at 42,000 cells/cm^2^ in 1% VIC medium, without antibiotics or antifungal supplements, and cultured overnight. We increased cell seeding density per the manufacturer’s recommendations for siRNA experiments. On day 1, *UTY* siRNAs and silencer negative control (Ambion, catalog no. AM4635) were incubated with Lipofectamine 3000 Transfection Reagent (Thermo Fisher Scientific, catalog no. L3000008) for 15 min at room temperature before adding it to fresh 1% VIC medium without antibiotics. The final siRNA concentration for the 12- and 25-mm hydrogels was 20 and 100 pmol, respectively. On day 2, the samples were monitored without medium change. On day 3, the samples were fixed for immunofluorescence and lysed for RNA collection. In total, *UTY* siRNA treatment lasted 48 hours.

### Immunofluorescence, imaging, and analysis

Cells were cultured on NP hydrogels for 48 hours before fixing with 4% (w/v) paraformaldehyde (Electron Microscopy Sciences, catalog no. 15710) for 20 min. Afterward, cells were permeabilized with 0.1% (w/v) Triton X-100 (Sigma-Aldrich, catalog no. 93443) for 1 hour. Permeabilization buffer was removed and 5% (w/v) bovine serum albumin (Sigma-Aldrich, catalog no. A8327) was added as blocking buffer overnight at 4°C. The following primary antibodies were diluted in blocking buffer and incubated for 1 hour at room temperature: αSMA (1:300; Abcam, catalog no. ab7817), RUNX2 (1:300; Abcam, catalog no. ab23981), methyl-lysine (MeK; 1:350; Novus Biologicals, catalog no. NB600824), and acetylated lysine (AcK; 1:350; Abcam, catalog no. ab190479). Samples were rinsed with 0.05% (v/v) Tween 20 (Sigma-Aldrich, catalog no. P1379) for 5 min. The following secondary antibodies were diluted in blocking buffer and incubated for 1 hour at room temperature protected from light: Alexa Fluor 488 goat anti-mouse (1:200; Thermo Fisher Scientific, catalog no. A11001), Alexa Fluor 647 goat anti-rabbit (1:200; Thermo Fisher Scientific, catalog no. A21245), or Alexa Fluor 647 donkey anti-rabbit (1:200; Thermo Fisher Scientific, catalog no. A32790). Nuclei were stained with 4′,6-diamidino-2-phenylindole (DAPI; 1:500; Roche, catalog no. 10236276001), and cytoplasm was stained with CellMask Orange (1:5000; Thermo Fisher Scientific, catalog no. H32713). Sample coverslips were rinsed with PBS and transferred to a glass-bottom well plate (CellVis, catalog no. P24-1.5H-N) for imaging on a Nikon Eclipse Ti2-E. Fluorescence was quantified after modifying a freely available MATLAB code (*28*), and fluorescence channel exposure was adjusted consistently in all conditions for representative image clarity. αSMA intensity ratios were calculated by normalizing αSMA intensity with CellMask cytoplasmic intensity. Nuclear RUNX2 intensity ratios were calculated by normalizing RUNX2 nuclear intensity to cytoplasmic intensity. Nuclear methylated lysine ratios were calculated by normalizing MeK nuclear intensity to cytoplasmic intensity. Nuclear acetylated lysine ratios were calculated by normalizing AcK nuclear intensity to DAPI intensity.

### RNA isolation and RT-qPCR

RNA was collected at specified time points using the RNeasy Micro Kit (QIAGEN, 74004) following the manufacturer’s protocol. Gels on 25-mm coverslips were inverted onto lysis buffer for 3 min and then rinsed with 70% (v/v) ethanol to collect lysed cellular material. After following the manufacturer’s protocol for RNA isolation, samples were analyzed for mRNA concentration using a NanoDrop 2000 spectrophotometer (Thermo Fisher Scientific). Sample concentrations were normalized to 1 ng/μl during cDNA preparation using the iScript Synthesis Kit (Bio-Rad, catalog no. 1708841), following the manufacturer’s protocol. To measure relative gene expression, iQ SYBR Green Supermix (Bio-Rad, catalog no. 1708882) was mixed with primer sequences found in table S2. Cycle threshold (Cq) values were determined using a CFX384 iCycler (Bio-Rad). All gene expression calculations were normalized to *RPL30* expression.

### Bulk RNA sequencing and analysis (porcine)

Before bulk paired-end RNA sequencing, RNA was isolated as described above. RNA quality was assessed by Agilent TapeStation 4200, and RNA quantity was determined by QuBit 3.0 Fluorometer. Approximately 200 ng of RNA per sample was used for sequencing. The Sanford Consortium for Regenerative Medicine’s Genomics Core conducted cDNA library preparation and indexing using the Illumina Stranded mRNA kit and IDT Illumina RNA UD Indexes, respectively. Sequences were checked for quality control using fastqc (v0.12.1) and subsequently removed adapter sequences using trimmomatic (v0.39) if adapter sequence content exceeded the threshold identified by fastqc. Alignment to the porcine genome (Sscrofa11.1) was conducted using HISAT2 (v2.2.1). Sequencing run statistics and genomic mapping accuracy are shown in table S3. Subsequent differential gene expression analysis was conducted in R (4.3.2) and R Studio (2023.12.1) using DESeq2 (Release 3.19) before we filtered for genes with a false discovery rate above 0.1. Up-regulated DEGs were identified by log_2_ fold change > 1, *P* value < 0.05, and false discovery rate < 0.1. Down-regulated DEGs were identified by log_2_ fold change < −1, *P* value < 0.05, and false discovery rate < 0.1. Figure S13 shows a heatmap of the most highly DEGs across each comparison. We used gene ontology analysis features in ingenuity pathway analysis (QIAGEN) for gene enrichment analyses of up-regulated and down-regulated genes in the porcine and human sequencing samples. Raw sequencing files are uploaded to National Center for Biotechnology Information (NCBI) Gene Expression Omnibus (GEO) repository and publicly available with accession number GSE273612. Gene counts for Y chromosome–linked genes *ZFY* and *KDM5D* confirmed biological sex of our male and female porcine mRNA samples.

### Single-cell sequencing, integration analysis, and statistical analysis (human)

We conducted a sex-separated reanalysis of previously published single-cell sequencing data for healthy patients and patients with calcific AV disease (*63*). We supplemented the analysis with two of our human valve sample patients diagnosed with AVS to increase biological replicates for each condition. Briefly, we prepared our human AV sample library using the Chromium Next GEM Single Cell 3′ Library Construction V3 Kit, following the Single Cell 3′ v3.1 Reagent Kits User Guide (10X Genomics, Document CG000204). We targeted 2000 to 5000 cells and sequenced at ~5000 reads per cell. The libraries were sequenced on the NovaSeq X Plus platform. Data were processed with Cell Ranger (v7.0.2), and raw counts were used to map the reads to the human reference genome (hg19). The outputted gene expression matrix was analyzed using the Seurat pipeline (v5.1.0) (*64*–*68*). We excluded cells with less than 200 unique genes or more than 5% mitochondrial transcripts. Overall, we analyzed 40,352 cells across six patients (tables S4 to S6).

We next applied Seurat’s NormalizeData function on the raw counts matrix; the function divides the feature counts of each cell by the total counts for that cell, scales that value by 10^6^, and applies a natural-log transformation. Next, we implemented the FindVariableFeatures function to choose the top 2000 highly variable genes using the “vst” selection method. After mean centering and scaling, we carried out principal components analysis on a merged gene expression matrix of all 40,352 cells. We used an elbow plot analysis to select 30 principal components as the cutoff dimension of our dataset. To batch correct for patient samples collected from different studies, we performed the Seurat single-cell RNA sequencing integration to match shared cell types across different datasets. To identify different cell types, we performed unsupervised clustering via the Louvain algorithm on *k*-nearest neighbors with the FindNeighbors and FindClusters (parameters *k* = 30 and resolution = 0.4) commands in Seurat. The functions returned eight cell clusters that were visualized on a UMAP. To label individual cell clusters, we performed a Wilcox test using the FindAllMarkers function in Seurat to plot the top 10 most DEGs (*P* value < 0.001 and log_2_ fold change > 1) for each cluster (fig. S3). Additionally, we used the top 10 conserved markers (minimum *P* value < 0.001 and log_2_ fold change > 0.5) across healthy and diseased samples to accurately label cell types (fig. S2). Gene names associated with epigenetic modifiers and calcification processes have been provided (tables S7 and S8). Gene expression was visualized using FeaturePlot, DoHeatMap, and VlnPlot functions in Seurat. Raw sequencing files are uploaded to NCBI GEO repository and publicly available with accession number GSE273980.

### Statistical analysis

For immunofluorescence studies, one-way analysis of variance (ANOVA) with Tukey’s posttests was conducted. We set a threshold of significance cutoff at *P* < 0.0001. Because of the experimental sample size (*n* > 1000 cells), we sought to reduce statistical bias by conducting a Cohen’s *d*-value test to determine significance independent of sample size. Thresholds of significance were represented by the following key: *d > 0.2, **d > 0.5, and ***d > 0.8 for between-sex significance; #*d* > 0.2, ##*d* > 0.5, and ###*d* > 0.8 for male-group significance; and $*d* > 0.2, $$*d* > 0.5, and $$$*d* > 0.8 for female-group significance. For RT-qPCR studies, one-way ANOVA with Tukey’s posttests were conducted. For Fig. 2, data are shown as the means ± SD, as calculated in GraphPad Prism.
