# Supplementary material for: Y chromosome–linked UTY modulates sex differences in valvular fibroblast methylation in response to nanoscale extracellular matrix cues
Source: Sci Adv. 2025 Mar 12;11(11):eads5717. doi: 10.1126/sciadv.ads5717 (PMC11900877; doi:10.1126/sciadv.ads5717)
Supplement: Supplementary file 1 — Figs. S1 to S13 Tables S1 to S8 Legend for data S1 [file sciadv.ads5717_sm.pdf]

Science Advances
AAAS

Supplementary Materials for

Y chromosome–linked UTY modulates sex differences in valvular fibroblast methylation in response to nanoscale extracellular matrix cues

Rayyan M. Gorashi *et al.*

Corresponding author: Brian A. Aguado, baguado@ucsd.edu

Sci. Adv. **11**, eads5717 (2025)
DOI: 10.1126/sciadv.ads5717

The PDF file includes:

Figs. S1 to S13
Tables S1 to S8
Legend for data S1

Other Supplementary Material for this manuscript includes the following:

Data S1

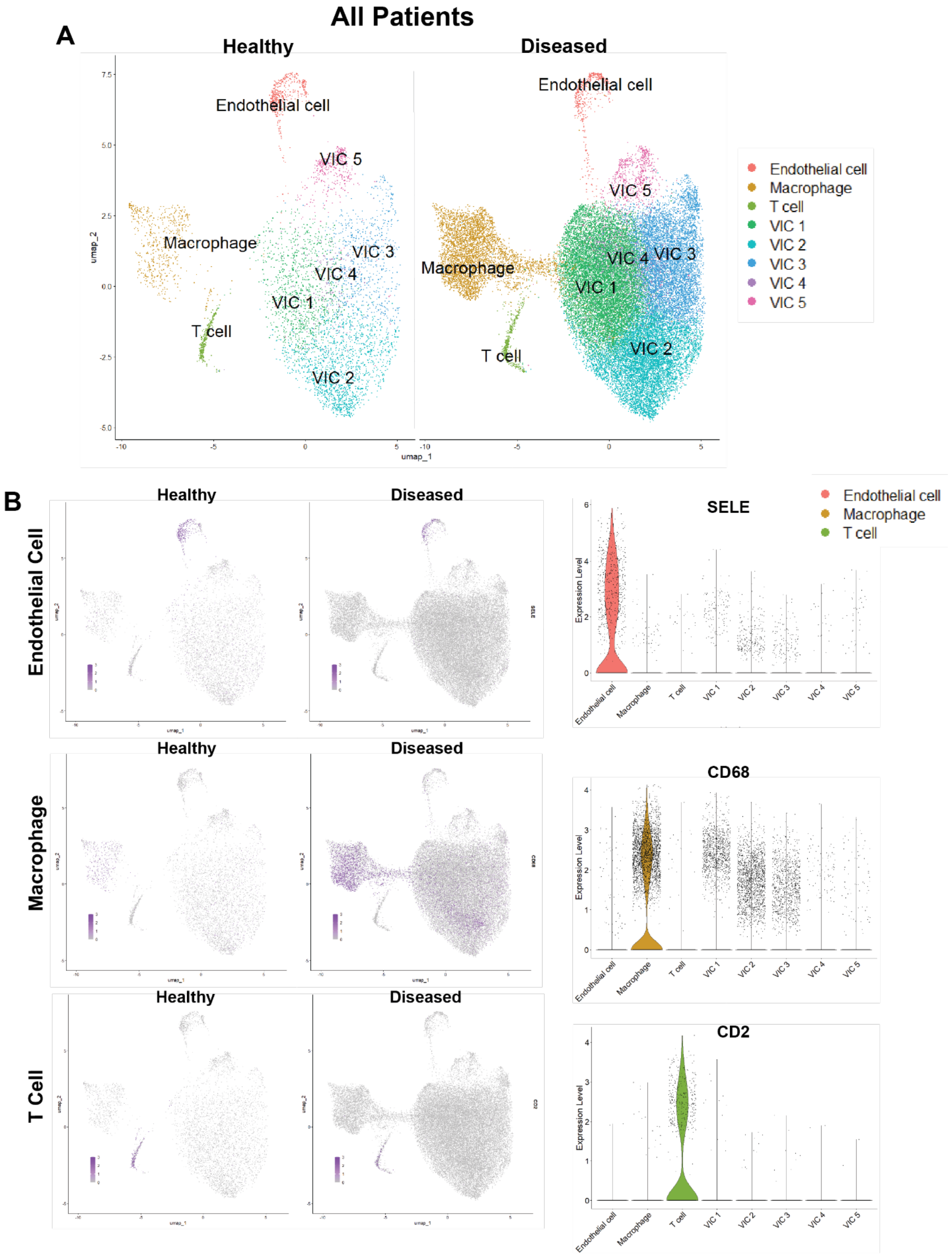

All Patients
A
Healthy
Diseased
Endothelial cell
Macrophage
T cell
VIC 1
VIC 2
VIC 3
VIC 4
VIC 5
umap_1
umap_2
B
Endothelial Cell
Macrophage
T Cell
SELE
CD68
CD2
Expression Level

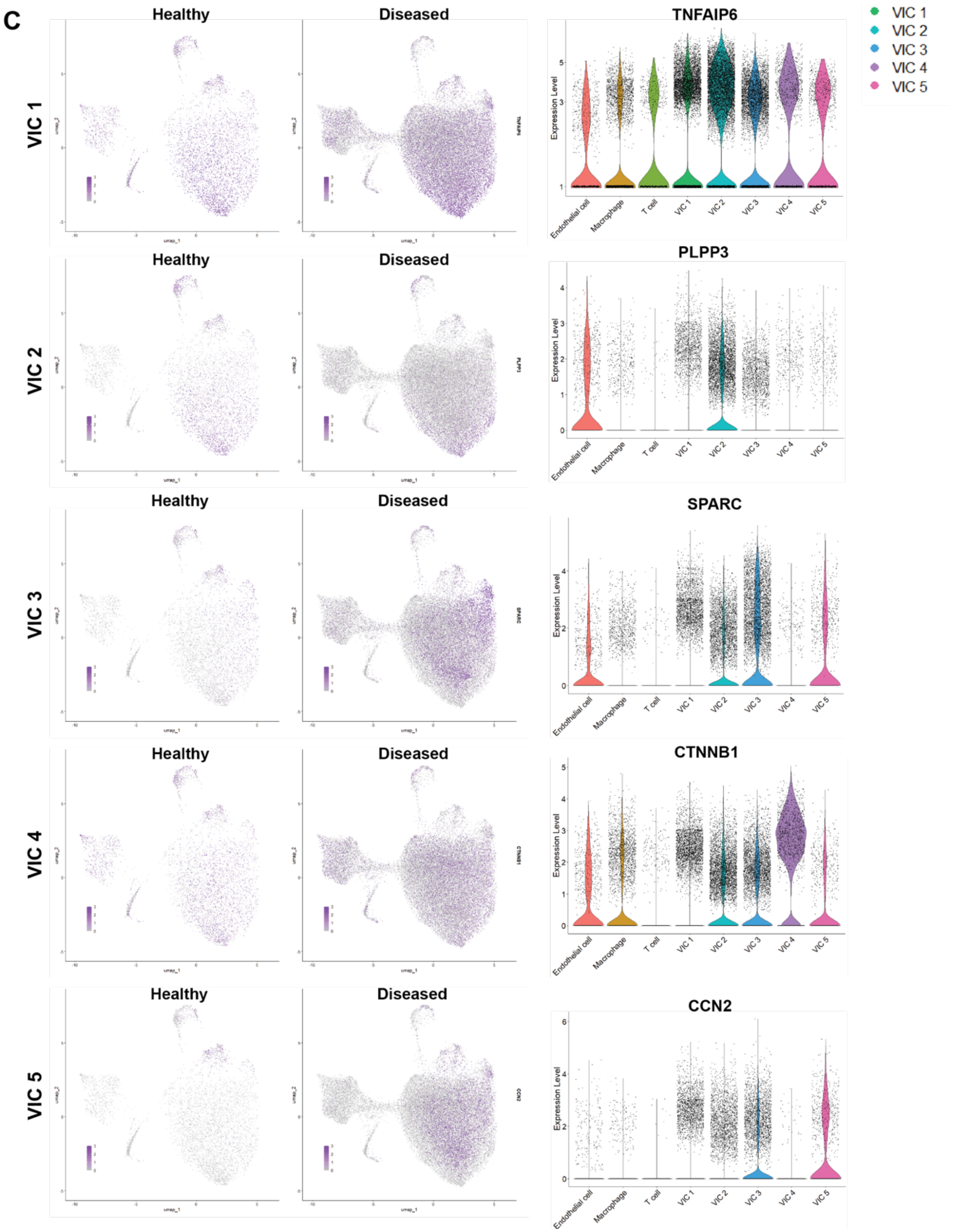

Figure S1: Human aortic valve cell type distribution and heterogeneity across sex and disease. (**A**) UMAP clustering and cell population distribution in healthy and diseased patient samples for males and females. Biomarkers used to identify distinct clusters in (**B**) endothelial cell and immune cell populations and (**C**) VIC populations.

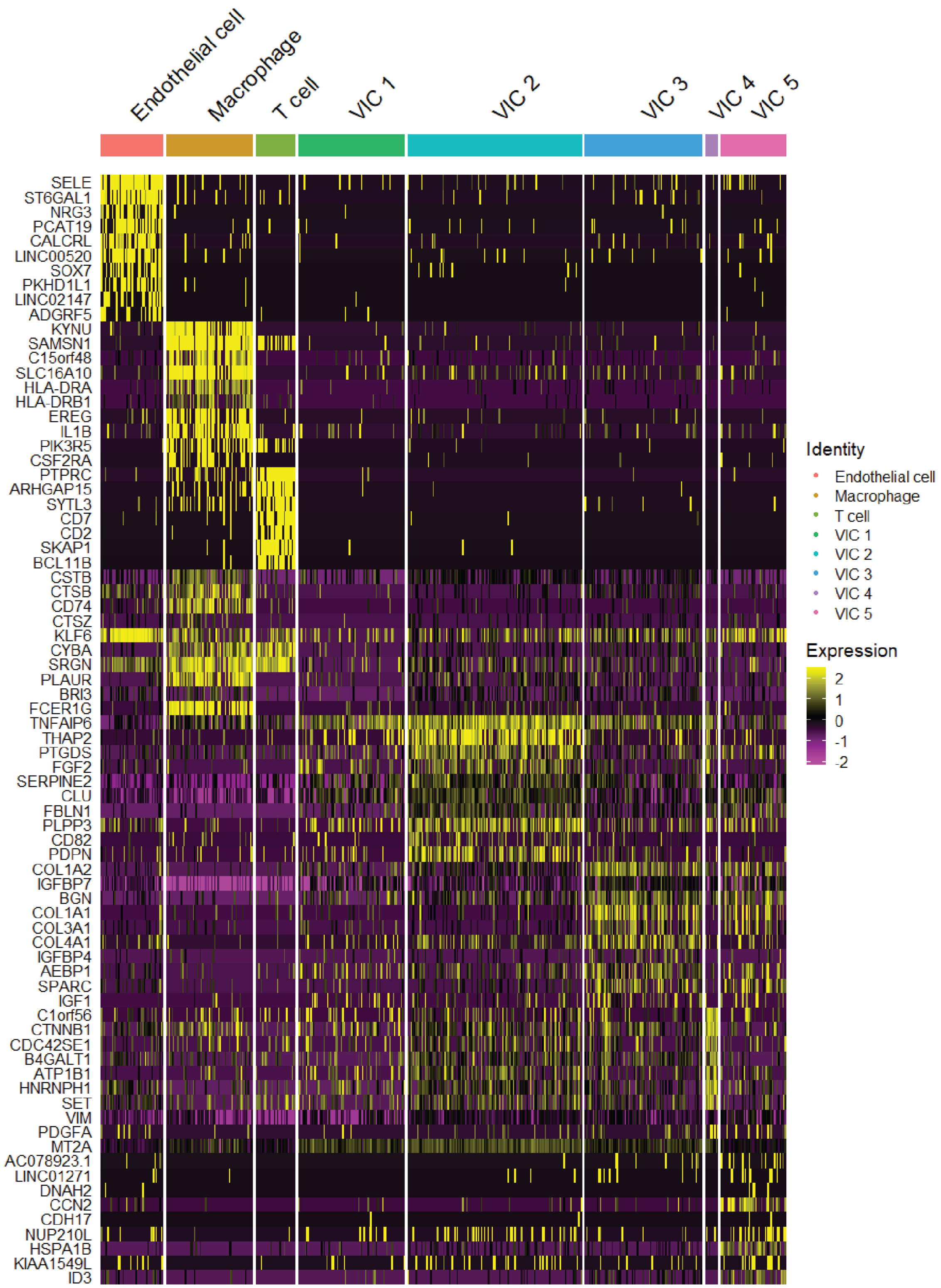

Figure S2: Heatmap of conserved biomarkers across all cell clusters for all patients.

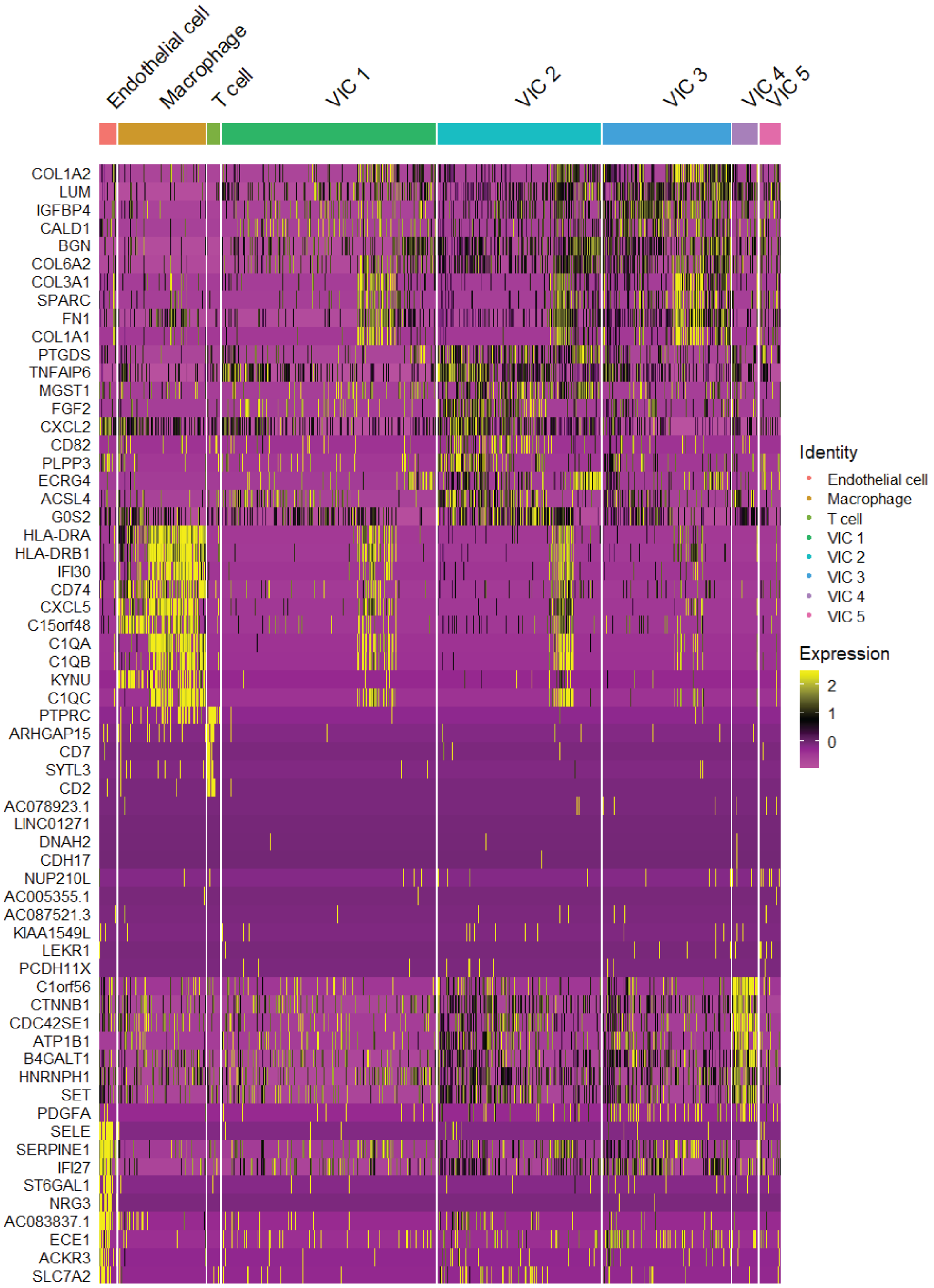

Figure S3: Heatmap of distinct biomarkers across all cell clusters for all patients.

A **Male VIC Populations**

	VIC/Total Cell Count	% *UTY+*	% *UTX+*
**VIC1 – Healthy**	24.02%	6.81%	6.60%
**VIC1 – Diseased**	37.11%	3.41%	5.96%
**VIC2 – Healthy**	32.20%	17.21%	17.50%
**VIC2 – Diseased**	25.23%	6.47%	9.42%
**VIC3 – Healthy**	9.24%	18.85%	17.14%
**VIC3 – Diseased**	20.98%	8.04%	11.93%
**VIC4 – Healthy**	4.86%	13.04%	9.78%
**VIC4 – Diseased**	4.99%	2.54%	2.93%
**VIC5 – Healthy**	6.23%	5.93%	7.63%
**VIC5 – Diseased**	1.52%	4.18%	6.11%

B **Female VIC Populations**

	VIC/Total Cell Count	% *UTY+*	% *UTX+*
**VIC1 – Healthy**	17.16%	0.00%	15.58%
**VIC1 – Diseased**	29.11%	0.00%	10.99%
**VIC2 – Healthy**	28.15%	0.00%	31.84%
**VIC2 – Diseased**	21.45%	0.00%	15.88%
**VIC3 – Healthy**	15.05%	0.00%	42.77%
**VIC3 – Diseased**	18.81%	0.00%	23.71%
**VIC4 – Healthy**	2.79%	0.00%	22.11%
**VIC4 – Diseased**	2.40%	0.00%	14.04%
**VIC5 – Healthy**	8.17%	0.00%	28.42%
**VIC5 – Diseased**	3.71%	0.00%	15.58%

Figure S4: Distribution of VIC populations in (A) male and (B) female patients, in healthy and diseased states.

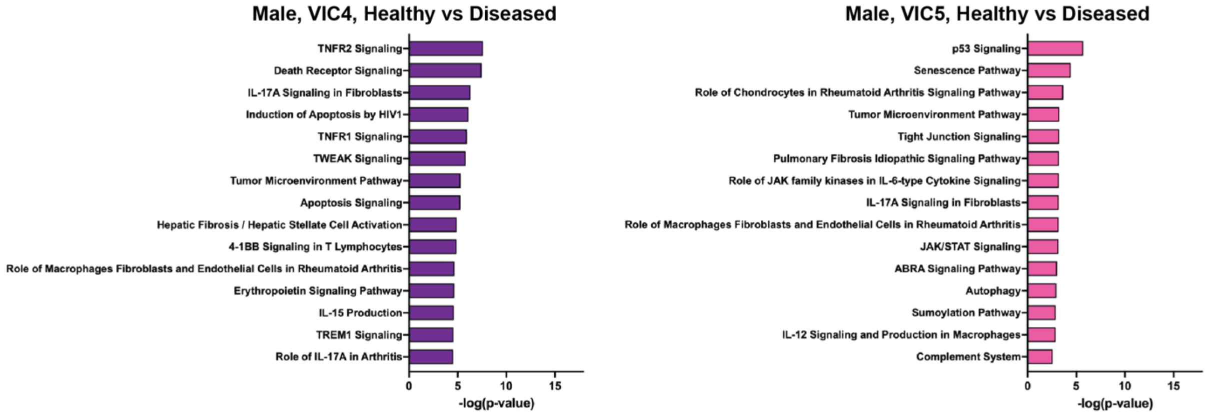

Figure S5: Ingenuity pathway analysis of male VIC4 and VIC5 populations.

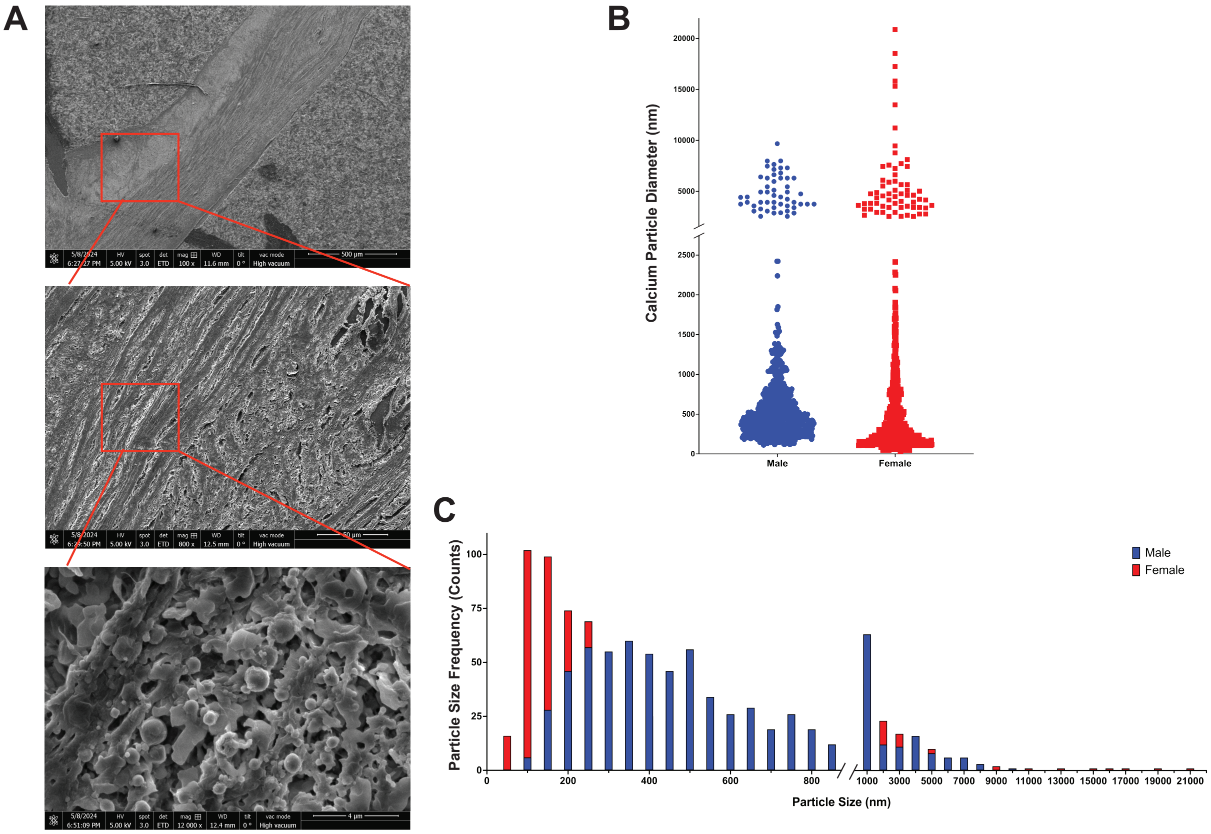

Figure S6: Quantification of particle size and distribution in diseased human aortic valve tissue. (**A**) SEM images of representative male aortic valve tissues to localize calcium phosphate particles. (**B**) Calcium particle diameter in diseased, age-matched male aortic valve tissue relative to female aortic valve tissue (n=2 male, n=2 female, n>600 particles, ****$p < 0.0001$). Diseased patients include mid and late stage AVS. (**C**) Particle diameter frequency distribution in male and female valve tissues (n=2 male, n=2 female, n>600 particles).

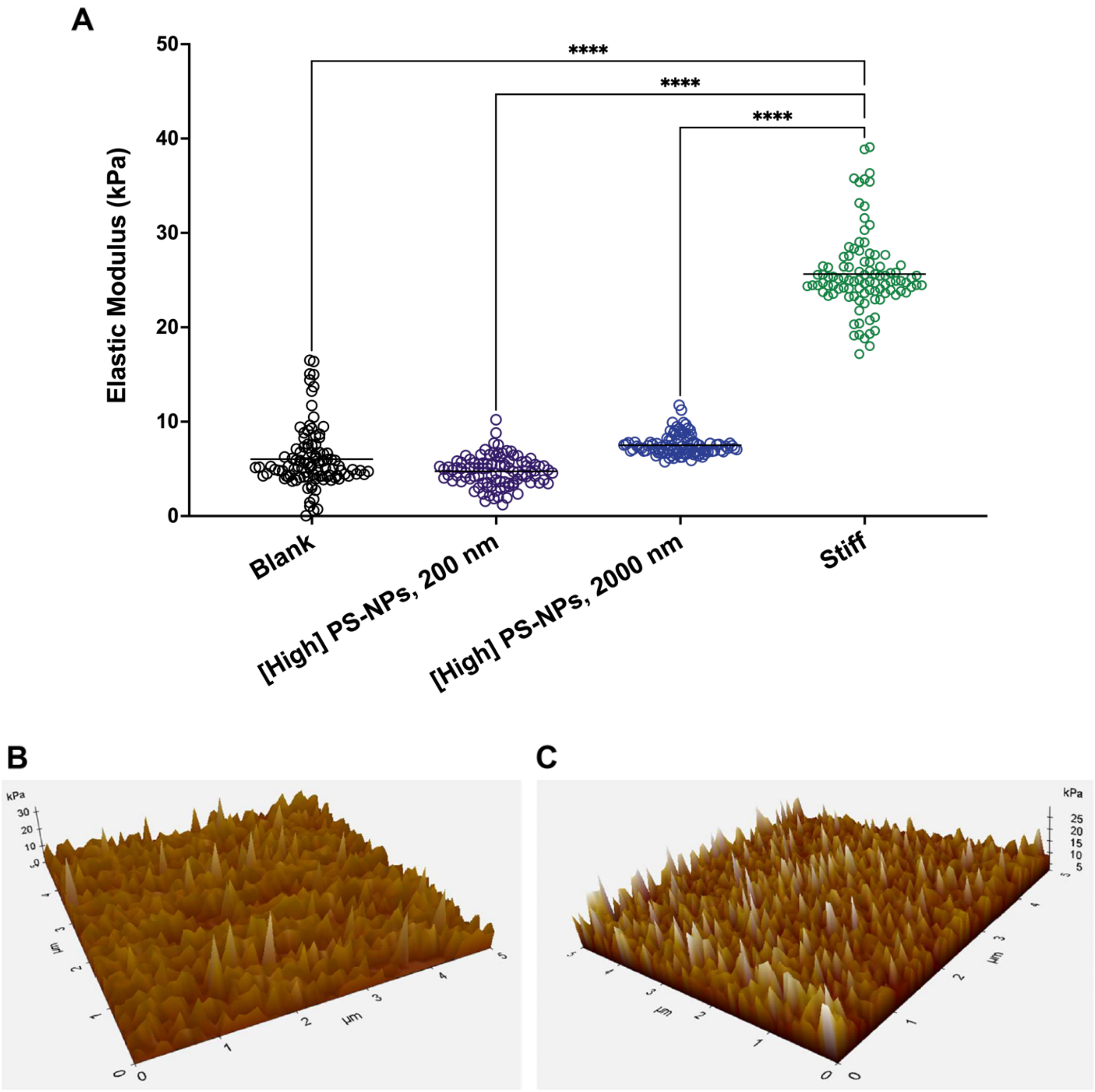

Figure S7: Atomic force microscopy (AFM) characterization of blank/soft and PS-NPs hydrogels. (**A**) Elastic moduli of blank/soft gels, blank gels with 200 nm PS-NPs, blank gels with 2000 nm PS-NPs, and stiff gels (n=50 measurements). Significance determined via one-way ANOVA (****$p<0.0001$ denoting significance between hydrogel formulations). (**B**) Representative atomic force microscopy (AFM) scan of blank/soft hydrogel modulus stiffness surface topography over a 5-μm^2 scan area. (**C**) Representative AFM scan of 2000 nm PS-NP hydrogel modulus stiffness surface topography over a 5-μm^2 scan area.

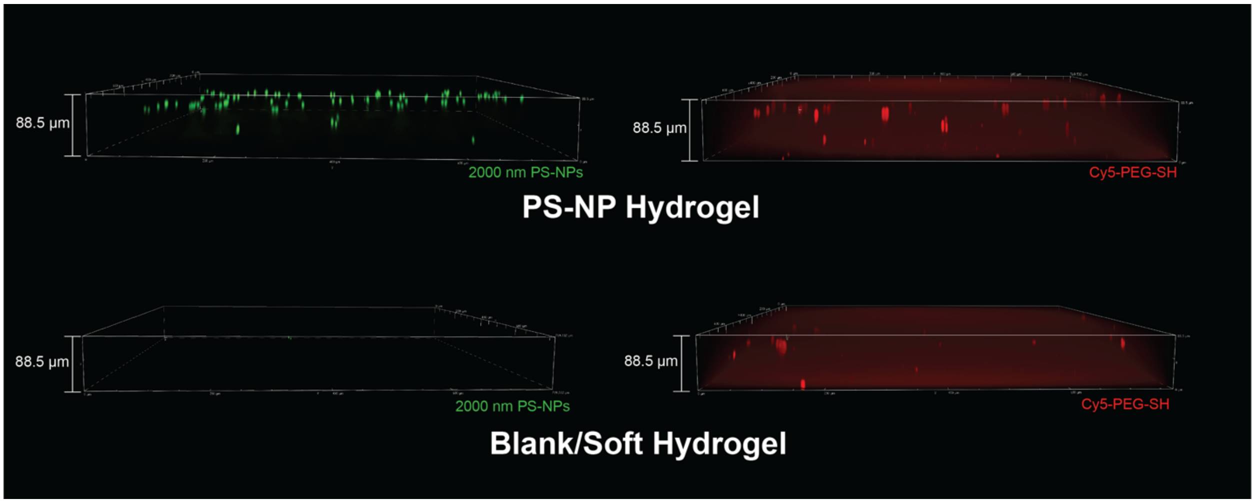

Figure S8: Three-dimensional rendered images of PS-NP and blank/soft hydrogel surface. The 2000 nm PS-NPs are visualized using fluorescent FITC in green, and the PEG-based hydrogel backbone is visualized with a fluorescent Cy5 thiol in red. PS-NPs are present across the surface of the PS-NP hydrogel (top) and absent in the blank/soft hydrogel (bottom).

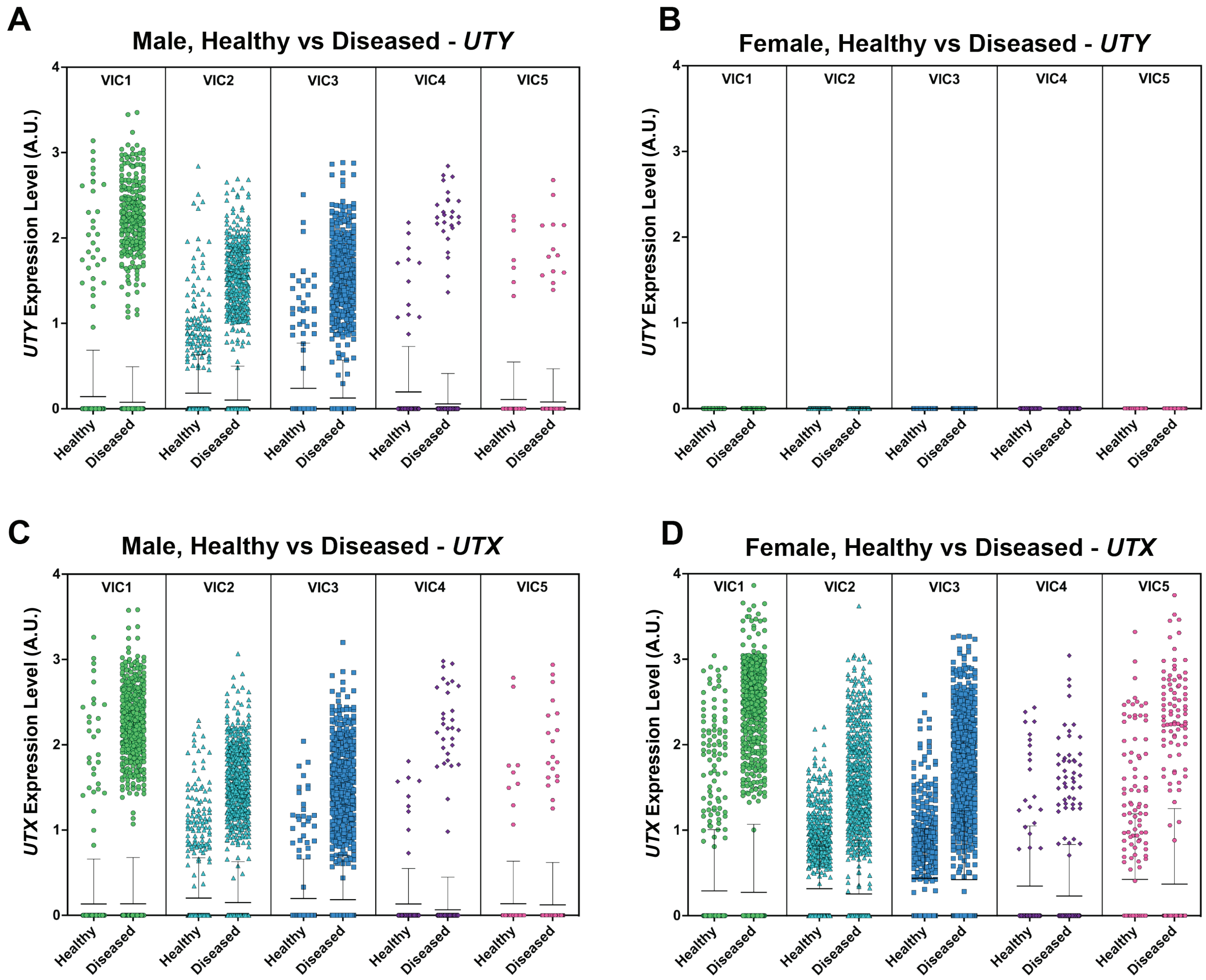

Figure S9: *UTY* and *UTX* mRNA expression in all VIC populations in patient samples. *UTY* relative mRNA expression in VIC populations 1 through 5 in (**A**) males and (**B**) females. *UTX* relative mRNA expression in VIC populations 1 through 5 in (**C**) males and (**D**) females.

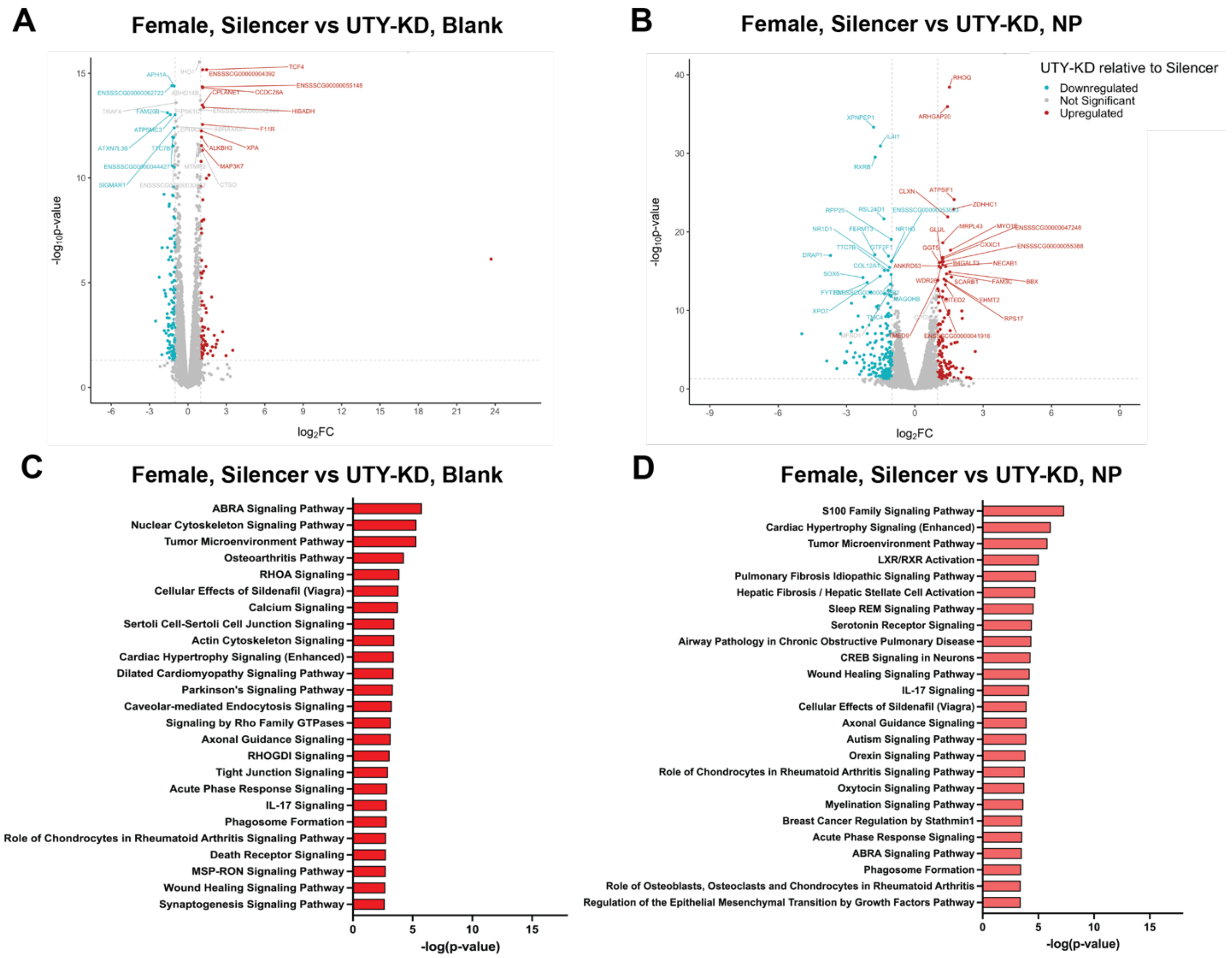

Figure S10. Bulk sequencing and ingenuity pathway analysis in female porcine VICs. Bulk mRNA sequencing shows differentially expressed genes in female VICs (blue = downregulated, red = upregulated, gray = not significant) in (**A**) blank and (**B**) PS-NP hydrogels using DESeq2 (n = 2 biological replicates). Associated pathways from ingenuity pathway analysis in female VICs in (**C**) blank and (**D**) PS-NP hydrogels.

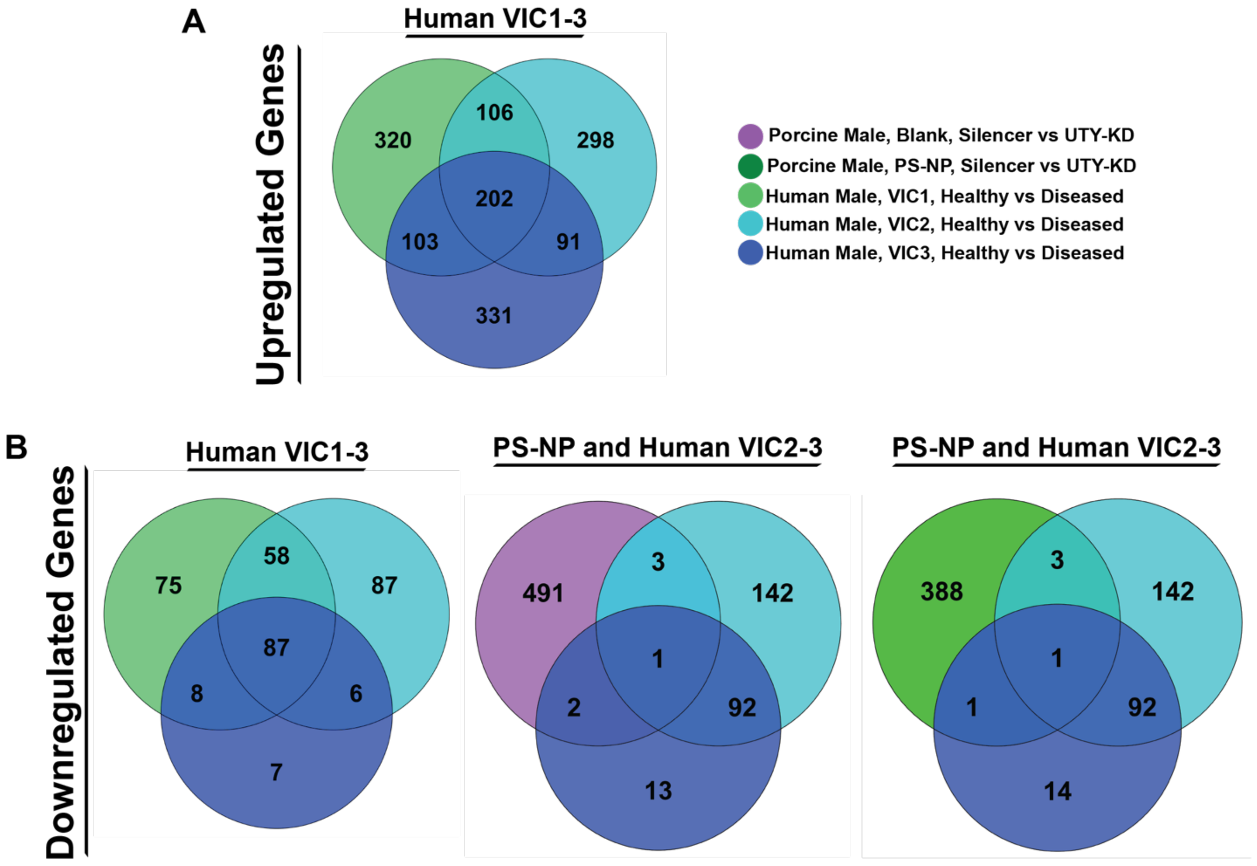

Figure S11: Venn diagrams of common genes across porcine and human sequencing sample comparisons (VIC1-3). (**A**) Venn diagrams of upregulated genes across conditions specified in legend. (**B**) Venn diagrams of downregulated genes across conditions specified in legend.

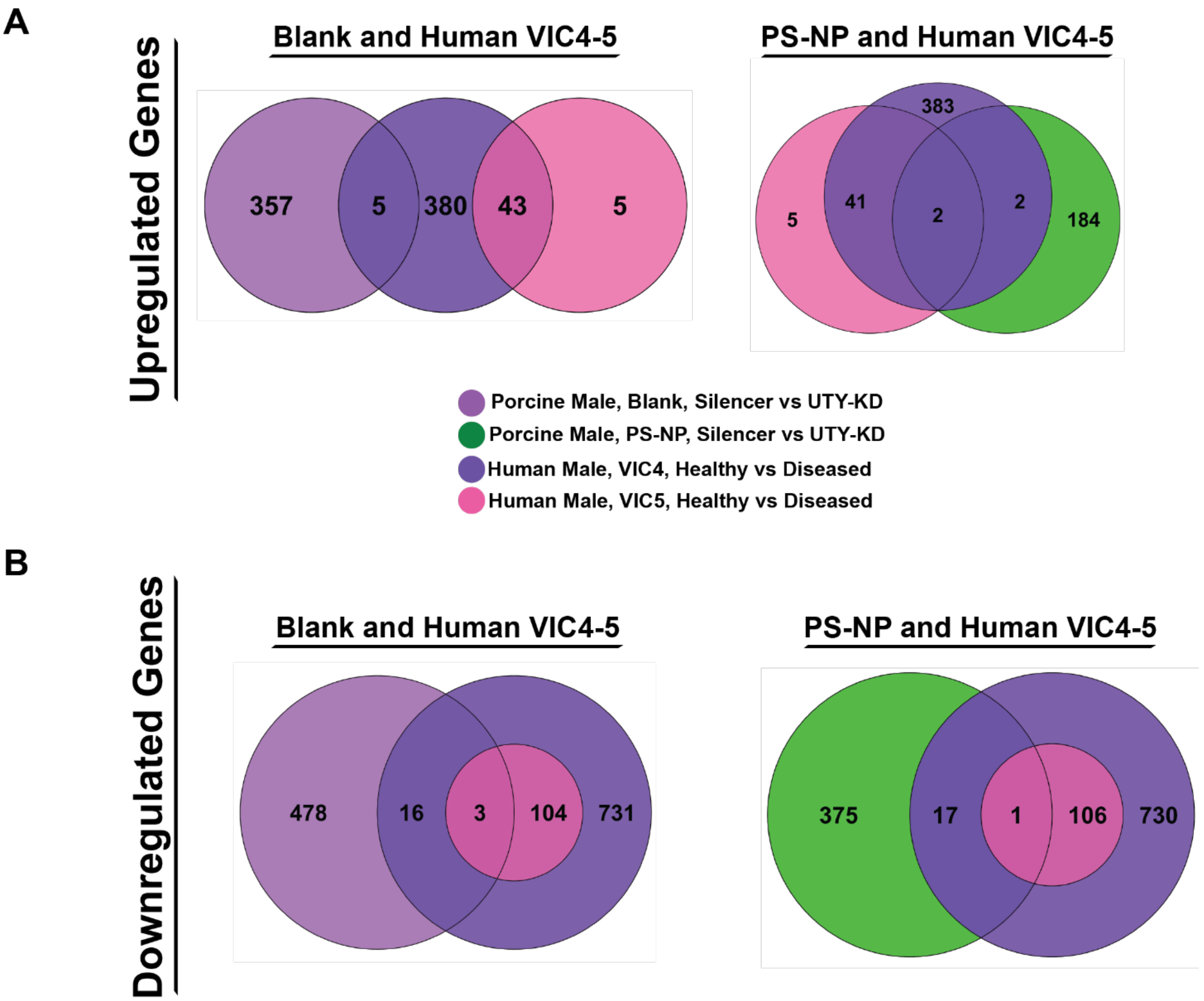

Figure S12: Venn diagrams of common genes across porcine and human sequencing sample comparisons (VIC4-5). (**A**) Venn diagrams of upregulated genes across conditions specified in legend. (**B**) Venn diagrams of downregulated genes across conditions specified in legend.

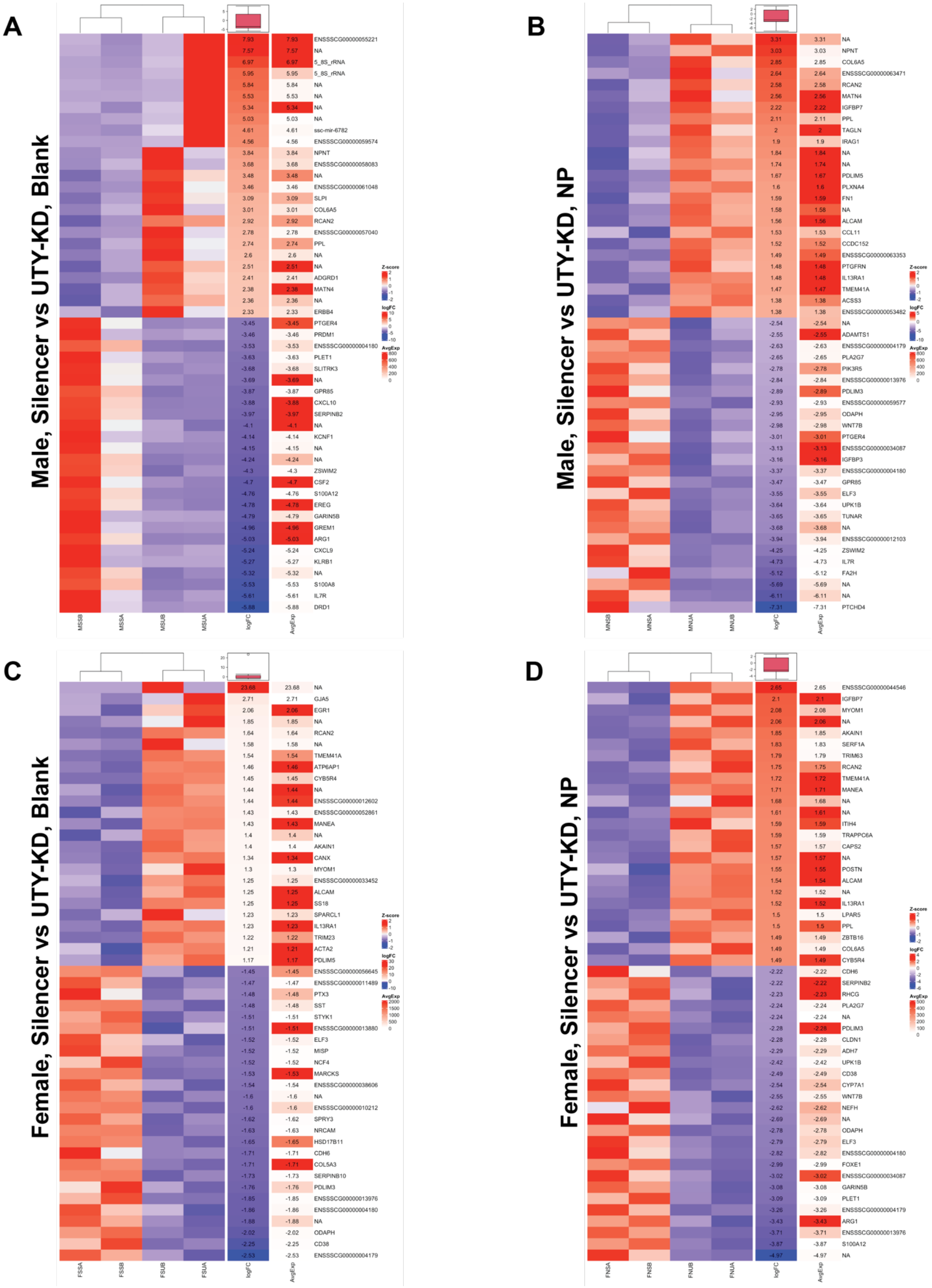
A
Male, Silencer vs UTY-KD, Blank
B
Male, Silencer vs UTY-KD, NP
C
Female, Silencer vs UTY-KD, Blank
D
Female, Silencer vs UTY-KD, NP

Figure S13. Heatmap of top differentially expressed genes after UTY-knockdown. Top differentially expressed genes (blue = downregulated, red = upregulated) after UTY-knockdown (UTY-KD) for male VICs on (**A**) blank hydrogels, (**B**) male VICs on PS-NP hydrogels, (**C**) female VICs on blank hydrogels, and (**D**) female VICs on PS-NP hydrogels.

Table S1. Human patient data.

Sample	Sex	Sample Type	Age	Comorbidities	NCBI GEO Data Accession Number
Patient 1	Male	Healthy	50	Unknown	PRJNA562645
Patient 2	Male	CAVD	50	Unknown	PRJNA562645
Patient 3	Male	AVS	79	Listed below[a]	GSE273980
Patient 4	Female	Healthy	48	Unknown	PRJNA562645
Patient 5	Female	CAVD	52	Unknown	PRJNA562645
Patient 6	Female	AVS	76	Listed below[b]	GSE273980

a) Cerebrovascular accident (CVA, Stroke), Congestive heart failure (CHF), Atrial fibrillation (Afib, A-fib, AF), Aortic (valve) stenosis, Chronic obstructive pulmonary disease (COPD), Pulmonary hypertension (pHTN, pulmonary HTN), Chronic kidney disease (CKD), Diabetes - Type II (T2D, T2DM), Hypothyroidism, Gastroesophageal reflux disease (GERD), Anemia, Thrombocytopenia, Coronary artery disease (CAD)

b) Atherosclerosis, Angina, Atrial fibrillation (Afib, A-fib, AF), Cardiac pacemaker, Pulmonary hypertension (pHTN, pulmonary HTN), Aortic (valve) stenosis, Chronic kidney disease (CKD), Heart Failure, Chronic obstructive pulmonary disease (COPD), Congestive heart failure (CHF), Coronary artery disease (CAD), Mitral (valve) stenosis, Anxiety disorder, Anemia, Emphysema, Diabetes - Type II (T2D, T2DM, Duration: 5 Years), Myocardial infarction (MI, heart attack), Cataract - bilateral, Arthritis

Table S2. RT-qPCR Porcine Primers.

Gene	Forward (5'-3')	Reverse (5'-3')
UTY	CACAATTGCAACTTTGTGCG	CGAATGTACCAACCCAGTCC
RPL30	AGATTTCCTCAAGGCTGGGC	GCTGGGGTACAAGCAGACTC

Table S3. Bulk RNA sequencing results statistics.

Sample	Number of Reads	% of >=Q30 Bases	Mean Quality Score	% of Raw Clusters	% of Perfect Index Reads
MSS-A	77,031,106	96.1	39.20	5.57	98.02
MSS-B	70,594,849	95.94	39.17	5.11	97.53
MSU-A	66,197,980	94.54	38.86	4.79	97.52
MSU-B	78,677,637	95.91	39.17	5.69	97.77
MNS-A	75,741,443	96.18	39.22	5.48	97.06
MNS-B	75,188,271	96.15	39.21	5.44	98.50
MNU-A	72,527,065	96.21	39.23	5.25	95.60
MNU-B	71,457,271	95.98	39.18	5.17	97.08
FSS-A	80,582,081	96.05	39.20	5.83	98.15
FSS-B	77,318,130	96.12	39.21	5.59	97.76
FSU-A	73,159,248	96.06	39.20	5.29	96.98
FSU-B	75,757,306	96.15	39.21	5.48	97.73
FNS-A	80,347,740	96.12	39.21	5.81	97.90
FNS-B	69,139,710	95.85	39.15	5.00	97.77
FNU-A	76,303,407	96.10	39.20	5.52	97.07
FNU-B	74,652,434	96.08	39.20	5.40	98.17

Table S4. Single cell sequencing results statistics.

Sample	Number of Reads	Mean Reads per Cell	Valid Barcodes	Q30 Bases in Barcode	Q30 Bases in RNA Read	Q30 Bases in Sample Index	Q30 Bases in UMI
Patient 3	41,797,983	6,562	96.7%	96.9%	96.0%	96.9%	97.9%
Patient 6	39,845,415	4,301	96.00%	97.10%	97.10%	97.10%	98.00%

Table S5. Single cell sequencing genomic mapping statistics.

Sample	Reads Mapped Confidently to Transcriptome	Reads Mapped Confidently to Exonic Regions	Reads Mapped Confidently to Intronic Regions	Reads Mapped Confidently to Intergenic Regions	Sequencing Saturation
Patient 3	96.1%	76.0%	12.4%	5.0%	49.7%
Patient 6	96.5%	74.1%	13.6%	4.8%	38.4%

Table S6. Single cell sequencing gene expression statistics.

Sample	Estimated Number of Cells	Fraction Reads in Cells	Mean Reads per Cell	Median Genes per Cell	Total Genes Detected	Median UMI Counts per Cell
Patient 3	6,370	88.4%	6,562	975	25,510	1,830
Patient 6	9,265	79.3%	4,301	693	25,733	1,166

Table S7. Epigenetic modifiers present in porcine and human data.

Sample	Epigenetic Modifiers
Porcine, Male, Blank Hydrogel, UTY-KD, Upregulated	TEX10, BRD3, NOC2L, SIRT2, CUL3, NEK9, SMC1A, BRPF3, PRKCB, SP1, SMARCC2, MSL1
Porcine, Male, PS-NP Hydrogel, UTY-KD, Upregulated	SUPT3H, ZNF687, ACTB, ATAD2B, DNMT3A, TAF6
Human, Male, Diseased, VIC1, Upregulated	SUPT3H, JAK2, ARID1B, BRWD3, L3MBTL4, KDM6B, PARG, FTO, SETD2, RAD54L2,CLOCK, PHIP, EYA3, NBN, MGA, NCOA1, ZMYM4, STK4, INO80D, SMARCAD1, NCOA2, MLLT6, ELP2, BTAF1, RNF168, TEX10, TET2, TRRAP, EYA4, TOP2B, MAP3K7, PHC3, ATR, NAT10, KAT6A, TET3, ZNF217, SENP1, TRIM33, KDM5A, ZMYM6, BRD8, CHEK1, APOBEC2
Human, Male, Diseased, VIC2, Upregulated	SUPT3H, DDX21, STK4, ARID1B, KDM6B, USP12, SMCHD1, L3MBTL4, PPP4R3B, PARG, NBN, ERCC6, CHUK, MAP3K7, BTAF1, CDK17, USP3, JAK2, SMARCAD1, BRWD3, HELLS, ALKBH1, TET3, DOT1L, PHF19, CHEK1, APOBEC2, ZNF217, TRIM24, KDM4A, ZNF711, JADE3, SCML2, RAD51
Human, Male, Diseased, VIC3, Upregulated	SUPT3H, DDX21, RNF168, SMARCAD1, ZMYM4, ARID1B, PARG, NBN, L3MBTL4, APOBEC2, TAF3, STK4, RBBP5, EXOSC6, SCMH1, USP36, SSRP1, ATAD2, CDK17, KDM6B, MLLT6, SETD2, ERCC6, USP11, USP15, BRWD3, RAD54L2, TAF1, SHPRH, EHMT1, CHUK, PRDM5, DNMT1, BAZ2A, ATR, INO80, REST, HELLS, CHEK1, BRD3, IKZF1, MAP3K7, SENP3, SETD1A, DZIP3, TYW5, JAK2, JADE3

Table S8. Gene names and abbreviations used.

Gene Name	Full Name
SUPT3H	SPT3 homolog, SAGA, and STAGA complex component
CCBE1	Collagen and calcium binding EGF domains 1
DEF6	FDCP 6 homolog
LRRN3	Leucine rich repeat neuronal 3
MBNL3	Muscle blind like splicing regulator 3
ROBO1	Roundabout guidance receptor 1
DGKH	Diacylglycerol kinase
UTY	Ubiquitously transcribed tetracopeptide repeat containing, Y-linked
UTX	Ubiquitously transcribed tetracopeptide repeat containing, X-linked

Data S1. (separate file)
Raw and quantified rheology, immunofluorescence, and RT-qPCR data for Figures 2-5.
